# Enhanced intersystem crossing in core-twisted aromatics†
†Electronic supplementary information (ESI) available: Includes details of the synthesis, structural information for all of the compounds (NMR, elemental analysis and mass spectra) and experimental details for the photophysical studies. CCDC 1402604 and 1402605. For ESI and crystallographic data in CIF or other electronic formats see DOI: 10.1039/c6sc05126j



**DOI:** 10.1039/c6sc05126j

**Published:** 2016-12-20

**Authors:** Kalaivanan Nagarajan, Ajith R. Mallia, Keerthi Muraleedharan, Mahesh Hariharan

**Affiliations:** a School of Chemistry , Indian Institute of Science Education and Research Thiruvananthapuram (IISER-TVM) , CET Campus, Sreekaryam , Thiruvananthapuram , Kerala 695 016 , India . Email: mahesh@iisertvm.ac.in ; Fax: +91-471-2597427

## Abstract

We describe the design, bottom-up synthesis and X-ray single crystal structure of systematically twisted aromatics **1c** and **2d** for efficient intersystem crossing. Steric congestion at the cove region creates a nonplanar geometry that induces a significant yield of triplet excited states in the electron-poor core-twisted aromatics **1c** and **2d**. A systematic increase in the number of twisted regions in **1c** and **2d** results in a concomitant enhancement in the rate and yield of intersystem crossing, monitored using femtosecond and nanosecond transient absorption spectroscopy. Time-resolved absorption spectroscopic measurements display enhanced triplet quantum yields (*Φ*_T_ = 10 ± 1% for **1c** and *Φ*_T_ = 30 ± 2% for **2d**) in the twisted aromatics when compared to a negligible *Φ*_T_ (<1%) in the planar analog **3c**. Twist-induced spin–orbit coupling *via* activated out-of-plane C–H/C=C vibrations can facilitate the formation of triplet excited states in twisted aromatics **1c** and **2d**, in contrast to the negligible intersystem crossing in the planar analog **3c**. The ease of synthesis, high solubility, access to triplet excited states and strong electron affinity make such imide functionalized core-twisted aromatics desirable materials for organic electronics such as solar cells.

## Abstract 2

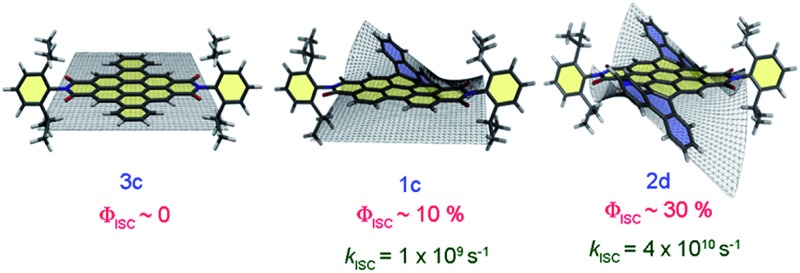
Core-twisted aromatics exhibit enhanced intersystem crossing upon photoexcitation when compared to their planar analogs.

## Introduction

**Scheme 1:**
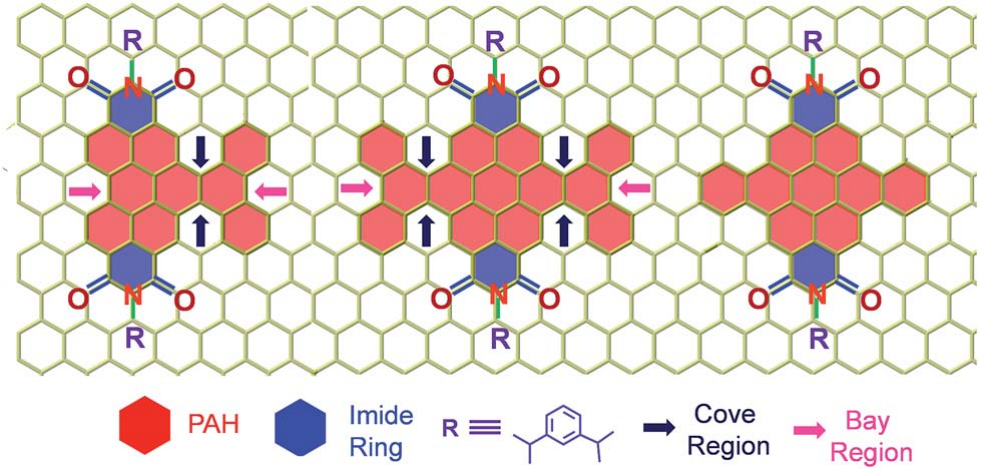
Molecular structure of the twisted derivatives **1c** and **2d** along with the planar derivative **3c** (arrows indicate the cove and bay regions in the derivatives).

Carbon based contorted nanostructures^1^ are emerging as vital components for optoelectronic devices,^2^ drug-delivery,^3^ catalysis^4^ and sensors.^5^ Planar nanostructures of carbon continue to attract immense interest for diverse applications^6^ despite a low singlet–triplet energy gap and weak spin–orbit coupling (SOC) diminishing intersystem crossing (ISC) in graphene.^7^ Enhancement of SOC in graphene has been achieved by dilute hydrogenation,^8^ fluorination,^9^ or proximity to WS_2_.^10^ Graphene grown on Cu, gold intercalated graphene grown on Ni,^11^ and Pb intercalated graphene grown on Ir^12^ show strong SOC (*ca.* 20–100 meV). Heavy adatoms (with partially filled p orbitals) deposited on a graphene lattice, also induce large intrinsic SOC.^13^ Hydrogenation of graphene generates non-planar sp^3^ sites that are responsible for the induced SOC, whereas other adatoms exhibit a heavy atom effect in promoting the ISC in graphenoid structures. Interestingly, curvature dependent excited state properties such as ISC were observed in fullerene derivatives.^14^ State-of-the-art theoretical and experimental investigations^15^ validate the importance of twists/nonplanarity in enhancing the SOC in graphenoid structures (Table S1 (ESI†)).^16^ Systematic incorporation of twists to activate ISC in heavy atom free^17^ sp^2^ hybridized graphenoid structures^18^ (Scheme 1) has received less attention.^7^ Though the effect of non-planarity on SOC is established in organic molecules,^19^ the phenomenon could not be generalized due to the observation of quantitative fluorescence in highly twisted chromophores.^20^

Our on-going interest in core-twisted^21^ organic chromophores^22^ prompted us to study the role of twisting in triplet formation. Recent efforts from our group on bay substitution with multiple bromine atoms revealed the core-twisted geometry of perylenediimide which results in an enhancement in triplet generation.^21^ Though core-twisting of perylenediimide through bay substitution is well established, the studies were not focused on the intersystem crossing properties of the materials.^23^ To isolate the influence of twisting from the heavy atom effect, it is imperative to impart heavy atom-free twisting in the chromophoric structure. Bottom-up approaches to synthesize non-planar aromatics,^24^ that include hexabenzocoronene,^25^ hexabenzoovalene,^26^ dibenzotetrathienocoronene,^27^ octabenzocircumbiphenyl,^28^ dimeric^29^ and core-twisted perylenediimides,^30^ are still of emerging interest for diverse opto-electronic applications. Hydrogen–hydrogen repulsion induced steric congestion at the cove region of the extended perylenediimide chromophore resulted in core-twisted aromatics **1c** and **2d** (Scheme 1) having π-extension lengths of 1.1–1.6 nm. The presence of imide in the nonplanar derivatives **1c** and **2d** improves (i) electron affinity;^31^ (ii) access to precisely functionalized edges^32^ and (iii) chemical/thermal/photochemical stability.^33^ We herein report the first systematic investigation on “twist-only” induced intersystem crossing (*k*_ISC_ = 1 × 10^9^ s^–1^ for **1c** and *k*_ISC_ = 4 × 10^10^ s^–1^ for **2d**) in imide functionalized core-twisted aromatics. Time-resolved absorption spectroscopic measurements display enhanced triplet quantum yields (*Φ*_T_ = 10 ± 1% for **1c** and *Φ*_T_ = 30 ± 2% for **2d**) in twisted aromatics when compared to a negligible *Φ*_T_ (<1%) in the planar analog **3c**.

## Results and discussion

### Synthesis and characterization

**Fig. 1:**
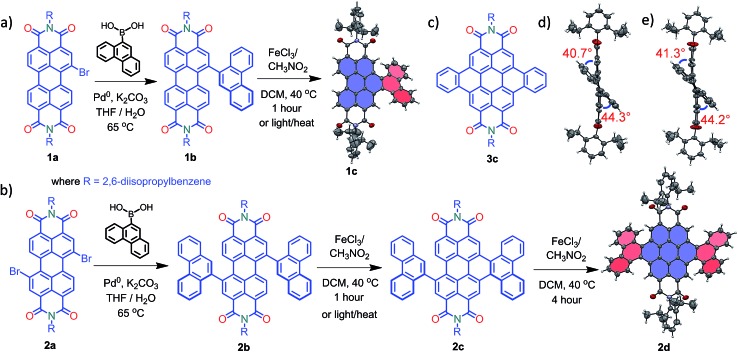
Synthetic route to the core-twisted aromatics (a) **1c** and (b) **2d**; (c) molecular structure of the planar analog **3c**; (d) and (e) twist angles at the cove regions in **1c** and **2d**.

Compounds **1c** and **2d** were synthesized *via* Suzuki coupling of one and two phenanthrene units, respectively, with perylenediimide (PDI) followed by the metal catalyzed Scholl dehydrogenation reaction (Fig. 1a and b). Bromination and imidisation of **1** were performed by following the procedure reported elsewhere.^21^ **1a** and **2a** were treated with one and two equivalents of 9-phenanthreneboronic acid to yield **1b** and **2b** respectively (ESI†). Further, **1b** and **2b** underwent the Scholl reaction with FeCl_3_ in dry DCM/CH_3_NO_2_ solution under nitrogen. Between 0–30 °C, cyclized products from **1b** and **2b** were formed in low yield (<1%). At a higher temperature (40 °C), the desired products **1c** and **2d** were obtained in 50% and 40% yields, respectively, under continuous nitrogen flow (Fig. S1–S4, ESI†). Intermediate **2c** (5% yield) was also isolated during the reaction and characterized using spectroscopic methods. A model planar derivative **3c** (Fig. 1c) was synthesized *via* Suzuki coupling of two benzene units with 1,7-dibromo PDI (**2a**) followed by the Scholl dehydrogenation reaction. Red fluorescent single crystals of **1c** and **2d** were obtained from chloroform and toluene solutions, respectively.

### Crystal structure analysis

**Fig. 2:**
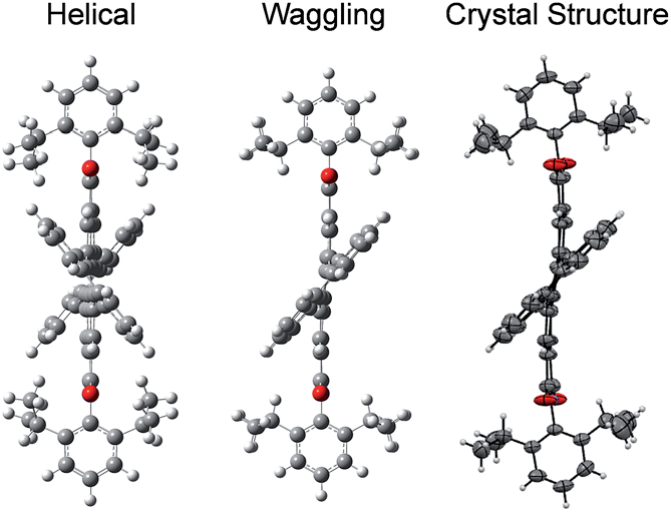
Possible helical and waggling conformations of the compound **2d**.

Compound **1c** crystallized in the tetragonal space group *P*4_3_, containing 4 molecules per unit cell (Table S2, ESI†). Repulsion between the H1′ and H8′ atoms of the phenanthrene unit and the H2 and H11 atoms of the PDI unit at the cove region (Fig. S5a, ESI†) resulted in twisting of the chromophore **1c**. The twist angle was calculated from the angle between the perylenediimide and phenanthrene planes in the crystal structure (Fig. S5b and d, ESI†). The twist angles at the two cove regions of compound **1c** were found to be 44.3° and 40.7° (Fig. 1d). Compound **2d** possessing a waggling^1^ conformation crystallized in the triclinic space group *P*1, with one molecule per unit cell (Table S2, ESI†). Repulsion between the H1′ and H8′ atoms of the phenanthrene units and the hydrogen atoms in the ortho region (H2, H5, H8 and H11) of PDI (Fig. S5c, ESI†) twist the chromophore **2d** at the 4 cove regions with a twist angle of 44.2° and 41.3° (Fig. 1e). To evaluate the thermodynamic stability between the helical and waggling^1^ (Fig. 2) conformations of the derivative **2d**, we conducted density functional theory (DFT) calculations at the B3LYP/6-311G++(d,p) level.^34^ From the DFT calculations, it is estimated that the waggling conformer of **2d** is thermodynamically more stable than the helical conformer by 17.5 kcal mol^–1^, which is in agreement with the waggling conformation obtained from the single crystal X-ray structure (Fig. 2). Single crystal X-ray structure analysis of **1c** and **2d** revealed a core-twisted polycyclic skeleton having 9 and 13 aromatic rings, respectively.

### Electrochemical properties

**Fig. 3:**
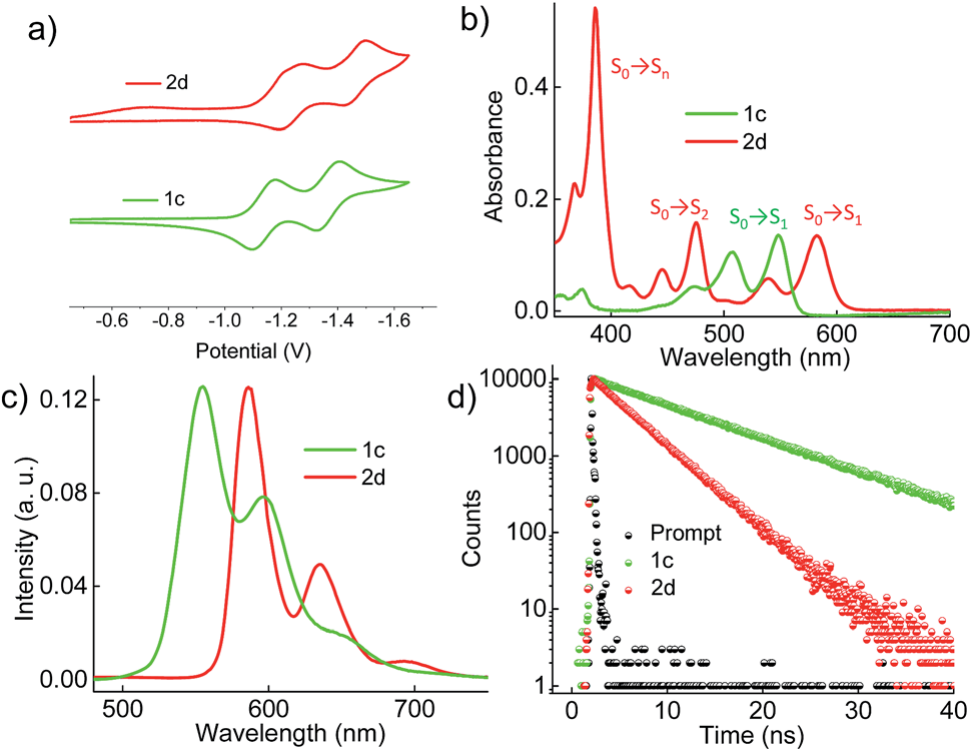
(a) Electrochemical measurement of the derivatives in dry DCM with reference to the Fc/Fc^+^ electrode, (b) UV-Vis absorption spectra, (c) emission (*λ*_ex_ = 480 nm), and (d) time-dependent fluorescence spectra (*λ*_ex_ = 480 nm and monitored at the respective emission maxima) of the derivatives **1c** and **2d** in toluene.

Cyclic voltammograms (0.1 M, *n*Bu_4_NPF_6_ in DCM) exhibited reversible reduction peaks (Fig. 3a) at –1.14 and –1.37 V for **1c**, and –1.22 and –1.46 V for **2d** (Table S3, ESI†) with reference to the Fc/Fc^+^ electrode. The reduction potentials of the derivatives **1c** and **2d** are more negative than those of the model derivative **3c** (–1.00 and –1.24 V), indicating that the **1c** and **2d** derivatives are significantly weaker electron acceptors. The highest occupied molecular orbitals (HOMOs) are distributed over the whole π system of the derivatives **1c**, **2d** and the model derivative **3c** (Fig. S6, ESI†). In contrast, the lowest unoccupied molecular orbitals (LUMOs) spread only at the coronenediimide core, due to the presence of electron withdrawing imide groups.

### Photophysical characterization

By virtue of their twisted nature, derivatives **1c** and **2d** with large π-surface dissolve well in common organic solvents like chloroform, dichloromethane and toluene. The UV-Vis absorption spectrum (Fig. 3b) of **1c** in toluene shows peaks centered at 475, 505 and 545 nm corresponding to π → π* (HOMO → LUMO) transitions (Table S4, ESI†). Derivative **2d** in toluene exhibits a π → π* transition at (i) 582 and 539 nm corresponding to S_0_ → S_1_ (HOMO → LUMO); (ii) 475, 445 and 416 nm corresponding to S_0_ → S_2_ (HOMO–1 → LUMO); and (iii) 385 nm corresponding to S_0_ → S_*n*_ (HOMO → LUMO+1), in agreement with DFT calculations (Fig. 3b). The model derivative **3c** exhibits peaks centered at 460 and 490 nm corresponding to π → π* (HOMO → LUMO) transitions as reported earlier.^35^ Upon excitation at 480 nm, **1c** shows vibronically resolved emission (Fig. 3c) centered at 555, 597 and 652 nm with a fluorescence quantum yield (*Φ*_f_) of 70%. Temperature dependent emission (*λ*_ex_ = 480 nm) and excitation (*λ*_em_ = 600 nm) spectra of **1b** in toluene indicated the evolution of a new species having fluorescence emission features identical to that of **1c** (Fig. S7a and b, ESI†). Spectroscopic analysis confirms the photocyclization of **1b** in toluene (*ca.* 1 μM) at higher temperature to yield **1c**. When compared to **1c**, derivative **2d** exhibits red-shifted emission centered at 586, 635 and 696 nm with a *Φ*_f_ value of 40%. The model derivative **3c** shows vibronically resolved emission centered at 510, 550 and 580 nm with a *Φ*_f_ value of 85% (Table S4 and Fig. S8a, ESI†). Partial reduction in the *Φ*_f_ values of **1c** and **2d** when compared with **3c** could be attributed to the non-radiative decay pathways arising from the nonplanar nature of the chromophores **1c** and **2d**.^16*a*^ Upon excitation at 480 nm, derivatives **1c** and **2d** in toluene exhibit fluorescence lifetimes (Fig. 3d) of 10 and 5.4 ns, respectively. The model derivative **3c** in toluene shows a monoexponential fluorescence lifetime of 5.5 ns upon excitation at 480 nm (Fig. S8b, ESI†).

### Nanosecond transient absorption measurements

**Fig. 4:**
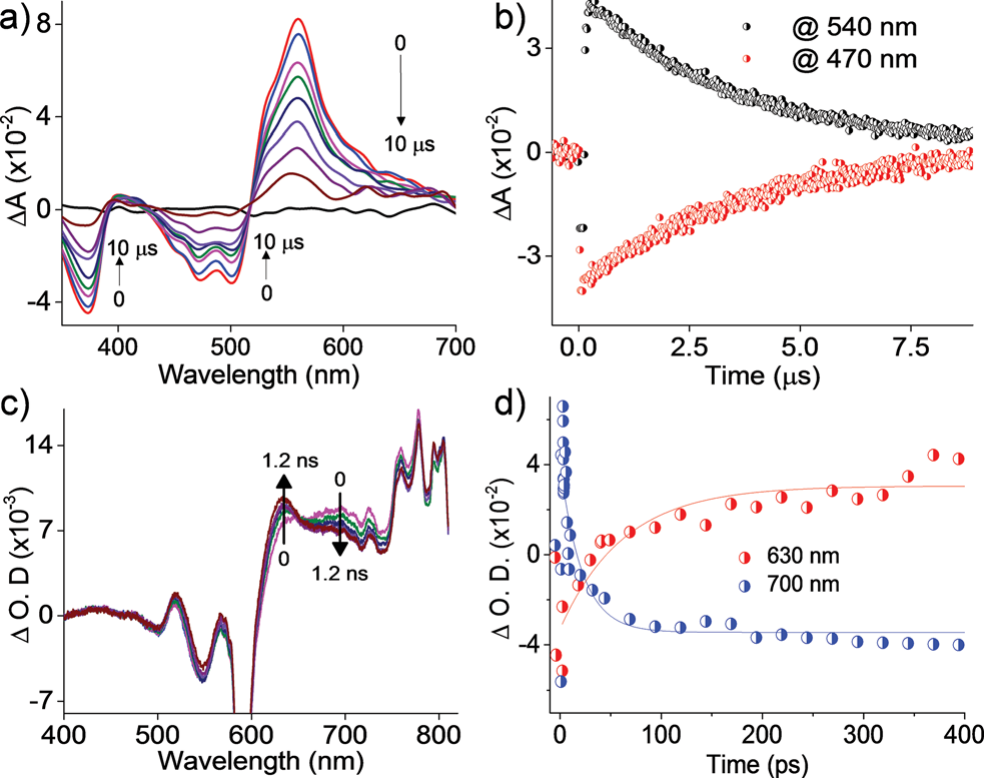
(a) nTA spectra and (b) corresponding decay profile of **1c** upon excitation at 355 nm in toluene; (c) fTA spectra and (d) corresponding decay profile of **1c** upon excitation at 300 nm in toluene.

Further insights into the excited state deactivation in core-twisted derivatives came from nanosecond and femtosecond transient absorption measurements. Upon excitation with a 10 ns laser pulse at 355 nm, **1c** in toluene (Fig. 4a) exhibited negative absorption peaks centered at 380, 470 and 510 nm corresponding to ground state depletion (S_0_ → S_*n*_). Due to the stronger positive signal at 520–620 nm, ground state bleaching from 520 to 570 nm is not seen in the nTA spectra compared to the UV-Vis absorption spectra. Observed twin absorption centered at 400 and 580 nm with a single exponential decay lifetime of 3.7 μs (Fig. 4b) is attributed to a triplet excited state in **1c**. Compound **2d** in toluene (Fig. S9a, ESI†) showed ground state depletion at 390, 480 and 590 nm, consistent with the UV-Vis absorption spectrum. Transient absorption corresponding to a triplet excited state is observed at 340, 420, 560 and 610 nm with a lifetime of 19.6 μs (Table S4 and Fig. S9b, ESI†). The existence of a triplet excited state in **1c** and **2d** was further confirmed by quenching of the transient spectra by oxygen purging. In contrast, the planar derivative **3c** in toluene exhibited negligible transient absorption upon excitation at 355 nm. Triplet quantum yields (*Φ*_T_) of **1c** and **2d** were calculated to be 10 ± 1% and 30 ± 2% (Table S4, ESI†), respectively, employing a triplet–triplet energy transfer method.^21^ Significant enhancement in the *Φ*_T_ values of **1c** and **2d** when compared to the model derivative **3c** is attributed to the twist induced SOC, as reported earlier.^19^ However, ISC was reported by Flamigni and coworkers in unsymmetrically substituted planar perylene derivatives which could be attributed to the nπ* to ππ* transition arising from bay imidisation.^36^ Attempts to record phosphorescence in the core-twisted derivatives **1c** and **2d** was not successful in DCM–EtOH glass at low temperature (77 K). However the derivatives **1c** and **2d** exhibited phosphorescence in DCM–EtOH–CH_3_I (1 : 1 : 0.1) glass at low temperature (77 K, Fig. S10, ESI†).

### Femtosecond transient absorption measurements

To unravel the kinetics of intersystem crossing, the core-twisted derivatives **1c** and **2d** in toluene were excited with a 110 fs laser pulse, at 300 nm. Femtosecond transient absorption (fTA) spectra of **1c** and **2d** showed a sharp negative absorption at 600 nm, corresponding to the second harmonic of the pump laser (2*λ*_ex_ = 600 nm). Photoexcitation of **1c** at 300 nm (Fig. 4c) displayed negative absorption at 505 and 545 nm along with positive absorption peaks centered at 510, 550, 630, 680 and 720 nm. Negative absorption observed at 505 and 545 nm could be attributed to ground state depletion consistent with the UV-Vis absorption spectrum. Singular value decomposition (SVD) of Δ*A versus* time and the wavelength based three-dimensional map of **1c** followed by global analysis yielded three principle components (Fig. S11, ESI†). Negative absorption centered at 560 nm with a lifetime of 9.5 ns is ascribed to stimulated emission. Positive absorption centered at 720 nm corresponds to S_1_ → S_*n*_ transitions that decay with a lifetime of 70 ps (*k*_IC_ = 0.14 × 10^11^ s^–1^; Fig. 4d). The rise time of an emerging positive absorption peak at 630 nm is estimated to be 1 ns (*k*_ISC_ = 1 × 10^9^ s^–1^) and is ascribed to a T_1_ → T_*n*_ transition. Upon excitation at 300 nm, **2d** in toluene (Fig. S9c, ESI†) showed ground state depletion at 475, 532 and 585 nm, consistent with the ground state absorption spectrum. SVD followed by global analyses of the positive absorption bands centered at 455, 516, 552 and 610–800 nm consist of three principal components (Fig. S12, ESI†). Negative absorption centered at 600 nm, with a lifetime of 5.1 ns is attributed to stimulated emission. The right singular vector at 720 nm decays with a lifetime of 6.5 ps (*k*_IC_ = 1.54 × 10^11^ s^–1^) and corresponds to a S_1_ → S_*n*_ transition (Fig. S9d, ESI†). During the decay centered at 720 nm, concomitant appearance of a new band at 630 nm is observed (Fig. S9c, ESI†). The emerging band at 630 nm with a rise time (*τ*_ISC_) of 25 ps (*k*_ISC_ = 4 × 10^10^ s^–1^) is attributed to a T_1_ → T_*n*_ transition in the derivative **2d**. According to the rates of internal conversion (*k*_IC_) and intersystem crossing (*k*_ISC_), the efficiency of ISC (*Φ*_ISC_ = *k*_ISC_/*k*_IC_) is calculated to be 7.1% and 26% for **1c** and **2d**, respectively, which is in agreement with the *Φ*_T_ calculated from the triplet–triplet energy transfer method. Quantum chemical calculations (Fig. S13, ESI†) indicate that out of plane C=C and C–H vibrations (*ν*_op_) can allow efficient ISC from a ππ* type singlet to a ππ* type triplet driven by Herzberg–Teller vibronic coupling in the core-twisted derivatives **1c** and **2d**.^37^

## Conclusions

In conclusion, we report the design and synthesis of solution processable electron deficient core-twisted aromatics, **1c** and **2d**. Femtosecond and nanosecond transient absorption measurements revealed “twist-only” induced ultrafast ISC in the non-planar derivatives **1c** and **2d**. Enhanced out of plane C=C and C–H vibrations facilitate efficient ISC with *Φ*_T_ values of 10 ± 1% and 30 ± 2% in the derivatives **1c** and **2d**, respectively, driven by Herzberg–Teller vibronic coupling. A higher *k*_ISC_ of 4 × 10^10^ s^–1^ for doubly twisted **2d**, when compared to a *k*_ISC_ of 1 × 10^9^ s^–1^ for singly twisted **1c**, clearly establishes the role of non-planarity in facilitating ISC in the reported derivatives **1c** and **2d**. Ease of solution processability and activated triplet excited states in the twisted aromatics **1c** and **2d** are beneficial for solar energy conversion by virtue of their long-lived triplet excited states. Current efforts in our laboratory are directed towards developing twisted chromophores for high performance opto-electronic devices.

## Experimental section

### Spectral measurements

Absorption spectra were recorded using a Shimadzu UV-3600 UV-VIS-NIR while emission (fluorescence/phosphorescence) and excitation spectra were performed using a Horiba Jobin Yvon Fluorolog spectrometer. All spectroscopic experiments were performed using standard quartz cuvettes with path length of 1 cm for solutions in dried and distilled solvents. The solution state fluorescence quantum yields were determined using optically matched solutions. Fluorescein dissolved in ethanol (*Φ*_f_ = 0.79)^38^ was used as a standard. *Φ*_f_ values for the samples were calculated as follows,
1

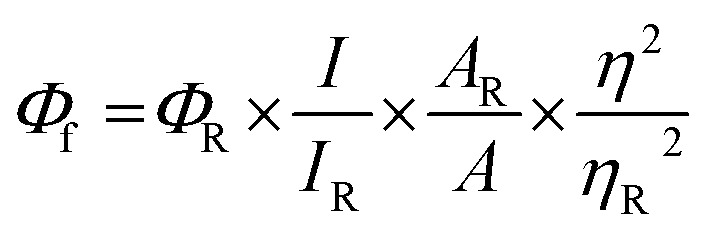




### Nanosecond transient absorption measurements

Laser flash photolysis experiments with nitrogen purged solutions were carried out using Applied Photophysics Model LKS-60 laser kinetic spectrometer with the second and third harmonic (355 nm and 532 nm, pulse duration ≈10 ns) of a Quanta Ray INDI-40-10 series pulsed Nd:YAG laser. Triplet states of the contorted aromatics **1c** and **2d** in toluene were confirmed using measurements of oxygen purged solutions through nanosecond flash photolysis studies. Triplet quantum yields^39^ upon direct photoexcitation (355 nm) were determined using [Ru(bpy)]Cl_2_ in methanol as standard (*Φ*_T_ = 1), with nonsaturating laser intensities. Equal volumes of a 0.2 mM solution of β-carotene were added to optically matched solutions of the reference and the sample. The equation for the triplet quantum yield is given by,
2

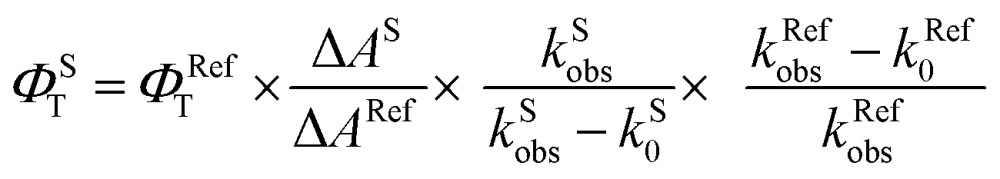

where, *Φ*ST and *Φ*RefT denote the triplet quantum yields of the sample and reference, respectively; Δ*A* ^S^ and Δ*A* ^Ref^ are transient absorption intensities of β-carotene in the sample and reference, respectively; *k*Sobs and *k*S0 are decay rates of the sample transient species before and after the addition of β-carotene. *k*Refobs and *k*Ref0 are decay rates of the reference transient species before and after the addition of β-carotene.

### Femtosecond transient absorption measurements

A Spectra-physics Tsunami Oscillator (80 MHz, 800 nm) was used as the seed for a Spectra-Physics Spitfire Regenerative amplifier (1 kHz, 4 mJ). A fraction of the amplified output was used to generate a 300 nm pump pulse. A residual 800 nm pulse was sent through a delay line inside an ExciPro pump-probe spectrometer from CDP Systems. A rotating CaF_2_ plate (2 mm thickness) was used to generate a continuum of white light from the delayed 800 nm pulse. The continuum of white light was split into two and the streams were used as probe and reference pulses. Transient absorption spectra were recorded using a dual diode array detector with a 200 nm detection window. Sample solutions were prepared in a rotating sample cell with a path length of 400 μm. IRF was determined by solvent (10% benzene in methanol) two photon absorption and was found to be approximately 130 fs at about 530 nm. Energy per pulse incident on the sample is attenuated, employing 80% neutral density filter when required. Toluene solution of the derivatives **1c** and **2d** were pumped with 300 nm, 200 nJ, ∼110 fs laser pulses and probed with the white light. Singular value decomposition (SVD) of Δ*A versus* time and a wavelength based three-dimensional map of the derivatives **1c** and **2d** was obtained from the fTA measurements. For SVD, the fTA spectra of **1c** and **2d** were constructed into a matrix using the Origin graphics software program (Version 8.5; MicroCal, Inc., Northampton, MA). Global analyses of the fTA spectra of the derivatives **1c** and **2d** were carried out using Glotaran (version 1.2).^40^

Efficiency of intersystem crossing (*Φ*_ISC_) could be estimated from the rates of internal conversion (*k*_IC_) and intersystem crossing (*k*_ISC_) as follows,^41^

3

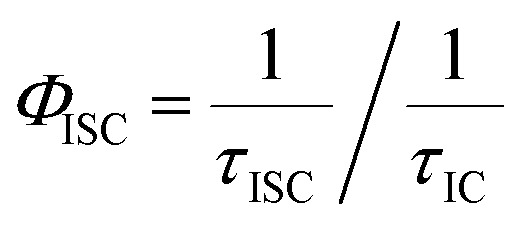

where, *τ*_ISC_ is the rate of intersystem crossing and *τ*_IC_ is the rate of internal conversion; extracted from the fTA spectra analysis.

### X-ray crystallography

High-quality specimens of appropriate dimensions were selected for the X-ray diffraction experiments. Crystallographic data collected are presented in the ESI.† Single crystals were mounted using oil (Infineum V8512) on a glass fibre. All measurements were made on a CCD area detector with graphite monochromated MoKα radiation. The data was collected using a Bruker APEXII detector and processed using an APEX2 from Bruker. All structures were solved by direct methods and expanded using Fourier techniques. The non-hydrogen atoms were refined anisotropically. Hydrogen atoms were included in idealized positions, but not refined. Their positions were constrained relative to their parent atom using the appropriate HFIX command in SHELXL-97. The full validation of CIFs and structure factors of **1c** and **2d** were performed using the CheckCIF utility and found to be free of major alert levels. 3D structure visualization and the exploration of the crystal packing of the derivatives were carried out using Mercury 3.1.

### Computational methods

Ground-state optimised structures and harmonic oscillator frequencies were computed using density functional theory (DFT) at the Becke's three parameter functional in combination with the Lee–Yang–Parr correlation functional (B3LYP) and 6-311++G(d,p) basis set. Vertical excitation energies and oscillator strengths were calculated employing time-dependent DFT (TD-DFT) at the B3LYP/6-311++G(d,p) level of theory. All computations were performed with the Gaussian 09 program suite.^34^
