# Supplementary material for: Enhanced intersystem crossing in core-twisted aromatics
Source: Chem Sci. 2016 Dec 20;8(3):1776–82. doi: 10.1039/c6sc05126j (PMC5477835; doi:10.1039/c6sc05126j)
Supplement: Supplementary file 1 [file SC-008-C6SC05126J-s001.pdf]

Electronic Supplementary Material (ESI) for Chemical Science.
This journal is © The Royal Society of Chemistry 2017

Enhanced Intersystem Crossing in Core-Twisted Aromatics

Kalaivanan Nagarajan, Ajith R. Mallia, Keerthi Muraleedharan and Mahesh Hariharan*

School of Chemistry, Indian Institute of Science Education and Research Thiruvananthapuram (IISER-TVM), CET Campus, Sreekaryam, Thiruvananthapuram, Kerala 695 016, Fax: (91)-471-2597427

Electronic Supplementary Information

S. No.	Contents	Page
1	Materials and Methods	2
2	Synthesis and Characterization	5
3	Table S1. A comparative account of the ISC properties (ΔE_{ST}, Φ_T and τ_{ISC}) of allotropes of carbon in various literature. Our data correspond to the core-twisted aromatics 1c and 2d.	12
4	Table S2. Single crystal analysis of 1c and 2d	13
5	Table S3. Electrochemical properties of the derivatives 1c, 2d and 3c	14
6	Table S4. Photophysical properties of the derivatives 1c, 2d and 3c	14
7	Figure S1. ^{1}H NMR spectrum of 1c	14
8	Figure S2. ^{13}C NMR spectrum of 1c	15
9	Figure S3. ^{1}H NMR spectrum of 2d	15
10	Figure S4. ^{13}C NMR spectrum of 2d	16
11	Figure S5. Chemical structures displaying H•••H repulsion in a) 1c and c) 2d; Schematic representation showing the perylenediimide and phenanthrene planes in the crystal structure of b) 1c and d) 2d.	16
13	Figure S6. Frontier molecular orbital diagram of a) 1c, b) 2d and c) 3c	17
14	Figure S7. Temperature dependent emission spectrum of dilute solution (1 x 10^{-6} M) of 1b in toluene (λ_{ex}=480 nm).	18
15	Figure S8. a) Absorption and emission (λ_{ex}=480 nm) spectra; b) time dependent fluorescence spectrum (λ_{ex}=480 nm; collected at 550 nm) of the model derivative 3c in toluene.	18
16	Figure S9. a) nTA spectra (λ_{ex}=355 nm); b) corresponding kinetics plots of excited singlet and triplet states in toluene; c) fTA spectra (λ_{ex}= 300 nm) and d) corresponding decay and bleach recovery profile of the compound 2d in toluene.	19
17	Figure S10. Normalized fluorescence spectra in DCM at RT and phosphorescence spectra in DCM-EtOH-CH_3I (1: 1: 0.1) glass at 77K of a) 1c and b) 2d.	19

18	Figure S11. fTA spectra of 1c in toluene (λ_{ex} = 300 nm) obtained from the SVD analysis (SE- stimulated emission; TA-triplet absorption; GSB - ground state bleach.	20
19	Figure S12. fTA spectra of 2d in toluene (λ_{ex} = 300 nm) obtained from the SVD analysis (SE- stimulated emission; TA-triplet absorption; GSB - ground state bleach.	20
20	Figure S13. a) Singlet and triplet energy levels of 1c, 2d and 3c calculated from TD-DFT at the basis set of BYLYP/6-311G++(d,p); b) comparison of low-lying singlet and triplet energy levels in the derivatives; c), d) and f) out of plane mode C-H bending in 1c, 2d and 3c respectively; g), h) and i) out of plane mode C=C bending in 1c, 2d and 3c respectively.	21

Materials and Methods:

All chemicals were obtained from commercial suppliers and used as received without further purification. All reactions were carried out in oven-dried glassware prior to use and wherever necessary, were performed under dry nitrogen in dried, anhydrous solvents using standard gastight syringes, cannulae, and septa. Solvents were dried and distilled by standard procedures. TLC analyses were performed on precoated aluminum plates of silica gel 60 F254 plates (0.25 mm, Merck) and developed TLC plates were visualized under short and long wavelength UV lamps. Flash column chromatography was performed using silica gel of 200-400 mesh employing a solvent polarity correlated with the TLC mobility observed for the substance of interest. Yields refer to chromatographically and spectroscopically homogenous substances. Melting points were obtained using a capillary melting point apparatus and are uncorrected. IR spectra were recorded on a Shimadzu IRPrestige-21 FT-IR spectrometer as neat thin films between NaCl plates in case of liquids and as KBr pellets in the case of solids. ^{1}H and ^{13}C NMR spectra were measured on a 500 MHz and 125 MHz Bruker advanced DPX spectrometer respectively. Internal standard used for ^{1}H and ^{13}C NMR is 1,1,1,1-tetramethyl silane (TMS). All CHN analyses were carried out on an Elementar vario MICRO cube Elemental Analyzer. All values recorded in elemental analyses are given in percentages. High Resolution Mass Spectra (HRMS) were recorded on an Agilent 6538 Ultra High Definition (UHD) Accurate-Mass Q-TOF-LC/MS system using either atmospheric pressure chemical ionization (APCI) or electrospray ionization (ESI) mode.

Spectral Measurements: Absorption spectra were recorded in Shimadzu UV-3600 UV-VIS-NIR while emission (fluorescence/phosphorescence) and excitation spectra were performed in Horiba Jobin Yvon Fluorolog spectrometer. All spectroscopic experiments were performed by using standard quartz cuvettes of path length 1cm for solution in dried and distilled solvents. The solution state fluorescence

quantum yields were determined by using optically matched solutions. Fluorescein dissolved in ethanol (Φ_{fl} = 0.79)[1] was used as the standard. The fluorescence quantum yield was calculated as follows,

$$\Phi = \Phi_R \times \frac{I}{I_R} \times \frac{A_R}{A} \times \frac{\eta^2}{\eta_R^2} \qquad (1)$$

Laser flash photolysis experiments of the nitrogen purged solutions were carried out in an Applied Photophysics Model LKS-60 laser kinetic spectrometer using the second and third harmonic (355 nm and 532 nm, pulse duration ≈10 ns) of a Quanta Ray INDI-40-10 series pulsed Nd:YAG laser. Triplet states of the contorted aromatics 1c and 2d in toluene were confirmed using the measurement of oxygen purged solutions through nanosecond flash photolysis studies. Triplet quantum yields[2] upon direct photoexcitation (355 nm) were determined by using $[Ru(bpy)]C1_2$ in methanol as standard (Φ_T = 1), with nonsaturating laser intensities. Equal volume of 0.2 mM solution of β-carotene was added to optically matched solutions of reference and the sample. The equation for the triplet quantum yield is given by,

$$\Phi_T^S = \Phi_T^{Ref} \, x \, \frac{\Delta A^S}{\Delta A^{Ref}} \, x \, \frac{k_{obs}^S}{k_{obs}^S - k_0^S} \, x \, \frac{k_{obs}^{Ref} - k_0^{Ref}}{k_{obs}^{Ref}} \qquad (2)$$

Where, Φ_T^S and Φ_T^{Ref} denote the triplet quantum yield of the sample and reference respectively; ΔA^S and ΔA^{Ref} are transient absorption intensity of β-carotene in sample and reference respectively; k_{obs}^S and k_0^S are decay rate of sample transient species before and after the addition of β-carotene. k_{obs}^{Ref} and k_0^{Ref} are decay rate of reference transient species before and after the addition of β-carotene. Spectra-physics Tsunami Oscillator (80 MHz, 800 nm) was used as seed for a Spectra-Physics Spitfire Regenerative amplifier (1 KHz, 4 mJ). A fraction of the amplified output was used to generate 300 nm pump pulse. Residual 800 nm pulse was sent through a delay line inside an ExciPro pump-probe spectrometer from CDP Systems. A rotating CaF_2 plate (2 mm thickness) was used to generate continuum of white light from the delayed 800 nm pulses. The continuum of white light was split into two and the streams were used as probe and reference pulses. Transient absorption spectra were recorded using a dual diode array detector with a 200 nm detection window. Sample solutions were prepared in a rotating sample cell with 400 μm path length. IRF was determined by solvent (10% Benzene in Methanol) two photon absorption and was found to be approximately 130 fs at about 530 nm. Energy per pulse incident on the sample is attenuated employing 80% neutral density filter when required. Toluene solution of the derivatives 1c, 2d were pumped with 300 nm, 200 nJ, ~110 fs laser pulses and probed by the white light. Singular value

decomposition (SVD) of ΔA versus time and wavelength based three-dimensional map of the derivatives 1c and 2d obtained from fTA measurements. For SVD, the fTA spectra of 1c and 2d were constructed into a matrix in the Origin graphics software program (Version 8.5; MicroCal, Inc., Northampton, MA). Global analyses of the fTA spectra of the derivatives 1c and 2d were carried out using Glotaran (version 1.2).[3]

Efficiency of intersystem crossing (Φ_{ISC}) could be estimated from the rates of internal conversion (k_{IC}) and intersystem crossing (k_{ISC}) as follows,[4]

$$\varphi_{ISC} = \frac{1}{\tau_{ISC}} \Big/ \frac{1}{\tau_{IC}}$$

Where, τ_{ISC} – rate of intersystem crossing and τ_{IC} – rate of internal conversion; extracted from the fTA spectra analysis.

X-ray Crystallography: High-quality specimens of appropriate dimensions were selected for the X-ray diffraction experiments. Crystallographic data collected are presented in the supplementary information. Single crystals were mounted using oil (Infineum V8512) on a glass fibre. All measurements were made on a CCD area detector with graphite monochromated Mo Kα radiation. The data was collected using Bruker APEXII detector and processed using APEX2 from Bruker. All structures were solved by direct methods and expanded using Fourier techniques. The non-hydrogen atoms were refined anisotropically. Hydrogen atoms were included in idealized positions, but not refined. Their positions were constrained relative to their parent atom using the appropriate HFIX command in SHELXL-97. The full validation of CIFs and structure factors of 1c and 2d were performed using the CheckCIF utility and found to be free of major alert level. 3D structure visualization and the exploration of the crystal packing of the derivatives were carried out using Mercury 3.1. Percentage (%) overlap was calculated from area of overlapped moieties of perylenediimide aromatic rings in the crystal structures. Percentage of overlapped aromatic area to total aromatic area gives the % overlap.[5]

Computational Methods: Ground-state optimised structure and harmonic oscillator frequencies were computed using density functional theory (DFT) at the Beake's three parameter functional in combination with the Lee-Yang-Parr correlation functional (B3LYP) and 6-311+G(d,p) basis set. Vertical excitation energies and oscillator strengths were calculated employing time dependent DFT

(TD-DFT) at the B3LYP/6-311+G(d,p) level of theory. All computations were performed with the Gaussian 09 program suite.[6]

Synthesis and Characterisation:

Synthesis of 1a and 2a

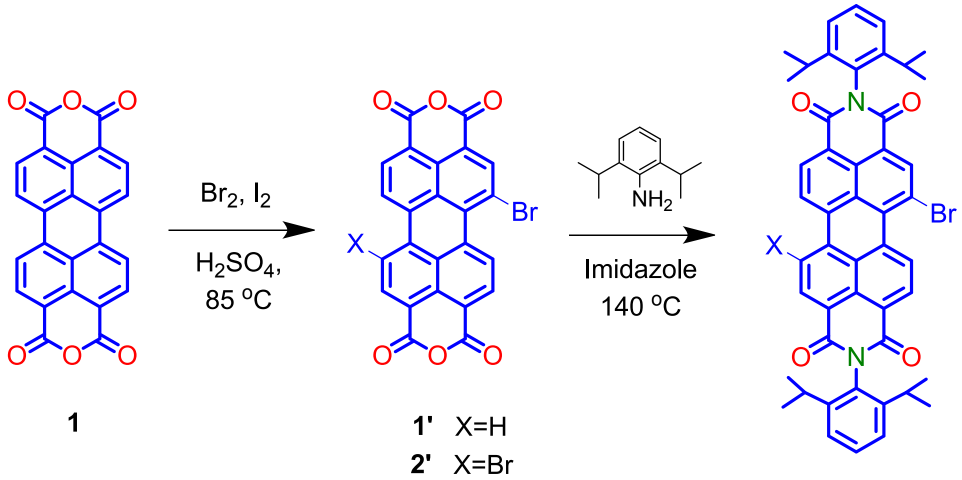

7.5 g of PTCDA (**1**) was stirred with 80 mL of concentrated sulphuric acid for 4 hours. 500 mg of elemental iodine was added and the reaction mixture was heated to 85°C. 1or 2 equivalents of elemental bromine was added drop wise to synthesise 1-bromo (**1'**) and 1,7-dibromo (**2'**) PTCDA respectively. The reaction mixture was refluxed for 24 hours. After 24 hours, the product was precipitated by pouring the reaction mixture into 200 mL of ice water. The product (PTCDA-Br_{1-2}) was filtered and dried in hot air oven. Since, the product was insoluble in any solvent, subjected to imidisation reaction without further purification. 1 g of **1'** / **2'** were mixed with 4 g of imidazole and 3 equivalents of 2,6-diisopropylaniline under nitrogen atmosphere. Reaction mixture was refluxed at 140 °C for 12 hours. After completion of the reaction, reaction mixture is poured into the solution of 60 % ethanol and 40 % 1N HCl. Red coloured precipitate was filtered and dried in hot air oven. **1a** and **2a** were purified by silica column chromatographic technique using dichloromethane/petroleum ether mixture (50:50) as the eluent.

1a (yield = 30 %). M.p. > 300 °C. ^{1}H NMR [500 MHz, $CDCl_3$, δ]: 9.69 (d, J= 8.00 Hz, 1H), 8.81 (s, 1H), 8.60 (m, 3H), 8.48 (d, J= 8.00 Hz, 2H), 7.50 (m, 2H), 7.33 (m, 4H), 2.72 (m, 2H), 2.56 (m, 2H)

1.12 (m, 6H), 1.10 (m, 6H), 1.06 (m, 6H), 1.05 (m, 6H). ^{13}C NMR [125 MHz, $CDCl_3$, δ]:170.4, 159.6, 140.4, 135.7, 133.0, 131.8, 131.2, 131.0, 129.4, 128.7, 127.3, 126.8, 124.7, 124.0, 60.8, 39.4, 26.4, 20.8. IR (KBr, cm^{-1}): 1735, 1693 and 1653. Elemental analysis: calcd. value for $C_{48}H_{41}BrN_2O_4$: 61.00% C, 3.76% H and 4.18% N; found: 61.15% C, 3.68% H and 4.14% N. HRMS (EI) m/z calculated for $C_{48}H_{41}BrN_2O_4$ $[M]^+$: 669.4749, found: 669.4735.

2a (yield = 70 %). M.p. > 300 °C. ^1H NMR [500 MHz, $CDCl_3$, δ]: 9.41 (d, J= 8.20 Hz, 2H), 8.84 (s, 2H), 8.62 (d, J= 8.20 Hz, 2H), 7.52 (m, 2H), 7.35 (m, 4H), 2.60 (m, 4H), 1.06 (m, 24H). ^{13}C NMR [125 MHz, $CDCl_3$, δ]: 171.09, 162.40, 138.11, 133.16, 132.98, 130.16, 129.31, 128.61, 126.98, 123.04, 122.62, 120.90, 62.20, 37.86, 27.34, 20.95. IR (KBr, cm^{-1}): 1733, 1698 and 1670. Elemental analysis: calcd. value for $C_{48}H_{40}Br_2N_2O_4$: 54.57% C, 3.23% H and 3.74% N; found: 54.68% C, 3.29% H and 3.61% N. HRMS (EI) m/z calculated for $C_{48}H_{40}Br_2N_2O_4$ $[M]^+$: 748.3710, found: 748.3704.

Synthesis of 1b:

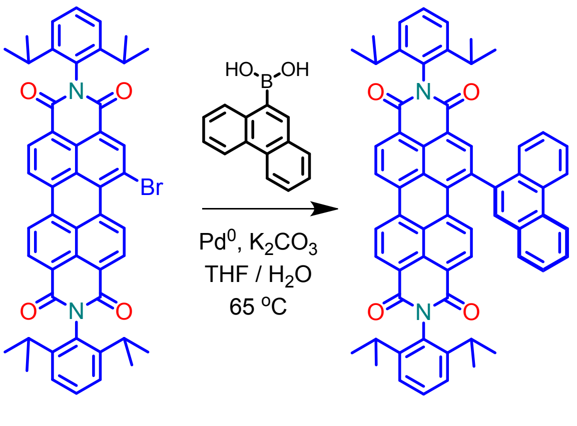

1a **1b**

0.2 mmol of 1-bromoperylenediimide (**1a**) was dissolved in dry THF and 10 mL of 2 M potassium carbonate was added. Reaction mixture was purged with nitrogen for 15 minutes. 0.02 mmol of tetrakis(triphenylphosphine)palladium(0) and 0.25 mmol of 9-phenanthreneboronic acid were added under nitrogen atmosphere. Reaction mixture was refluxed at 65 °C under nitrogen atmosphere. After completion of the reaction, product was extracted in dichloromethane and purified by column chromatography using dichloromethane/petroleum ether mixture (60:40) as eluent. Red coloured solid was obtained as product (**1b**) in 50 % yield. M.p. > 300 °C. ^1H NMR [500 MHz, $CDCl_3$, δ]: 8.82 (m, 4H), 8.73 (s, 2H), 8.66 (s, 1H), 8.02 (d, 1H), 7.85 (m, 3H), 7.66 (m, 4H), 7.40 (m, 3H), 7.28 (t, 2H), 7.19

(d, 2H), 2.73 (m, 2H), 2.56 (m, 2H), 1.15 (m, 6H), 1.13 (m, 6H), 1.06 (m, 6H), 1.05 (m, 6H).^{13}C NMR [125 MHz, $CDCl_3$, δ]: 163.67, 163.56, 163.29, 163.23, 145.71, 145.62, 145.50, 139.98, 139.12, 137.53, 135.57, 135.06, 134.96, 134.27, 131.83, 131.71, 131.62, 131.49, 131.44, 130.50, 130.47, 130.39, 129.75, 129.69, 129.07, 128.97, 128.22, 127.76, 127.71, 127.58, 127.55, 127.41, 127.13, 126.56, 125.55, 124.16, 124.07, 123.83, 123.40, 123.34, 123.05, 122.98, 122.48, 122.41, 31.94, 29.71, 29.27, 29.24, 29.13, 29.11, 24.07, 24.00, 23.97, 23.92, 23.91, 22.71. IR (KBr, cm^{-1}): 2960, 1705, 1668, 1591, 1497, 1458, 1339 and 1250. Elemental analysis: calcd. value for $C_{62}H_{50}N_2O_4$: 83.95% C, 5.68% H and 3.16% N; found: 84.05% C, 5.50% H and 3.15% N. HRMS (EI) m/z calculated for $C_{62}H_{50}N_2O_4$ $[M]^+$: 886.3771, found: 886.3762.

Synthesis of 1c:

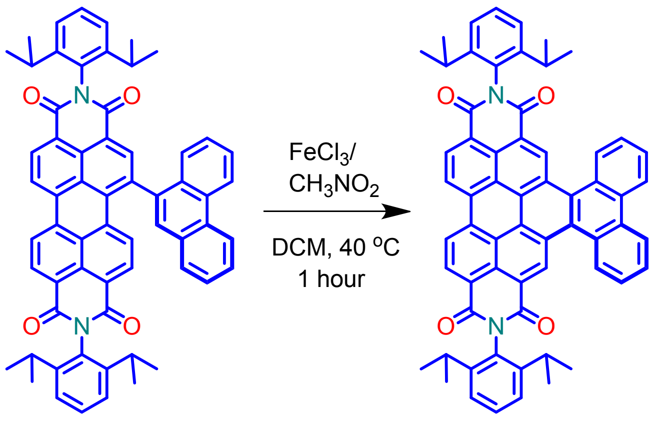

1b **1c**

100 mg of **1b** was dissolved in 500 mL of dry dichloromethane and heated to 40 °C under nitrogen atmosphere. A solution of 8 equivalents ferric chloride in 10 mL dry nitromethane was added dropwise into the reaction mixture under continuous nitrogen purging. The reaction mixture was refluxed at 40 °C for 1 hour. Reaction temperature was limited by the boiling point of dichloromethane. Completion of the reaction is monitored through thin layer chromatographic (TLC) technique. Then methanol was poured into the reaction mixture to stop the reaction. Reddish orange coloured product was precipitated. **1c** was purified by silica column chromatographic technique using dichloromethane/petroleum ether (60:40) mixture as eluent. Yield obtained was 50 %. Compound **1c** crystals were obtained from the slow evaporation of chloroform solution. M.p. > 300 °C. ^{1}H NMR [500 MHz, $CDCl_3$, δ]: 10.30 (s, 2H), 9.41 (d, 2H), 9.15 (d, 2H), 8.77 (d, 2H), 8.64 (d, 2H), 7.78 (m, 4H), 7.46 (t, 2H), 7.32 (d, 4H), 2.83 (m, 4H),

1.16 (m, 24H). ^{13}C NMR [125 MHz, $CDCl_3$, δ]: 164.20, 164.15, 145.77, 134.95, 134.11, 131.70, 131.03, 130.82, 129.83, 129.68, 128.75, 128.54, 128.47, 128.07, 127.64, 126.68, 124.93, 124.74, 124.15, 123.43, 122.46, 122.00, 53.41, 29.70, 29.32, 24.09, 24.05. IR (KBr, cm^{-1}): 2960, 1709, 1670, 1595, 1458, 1327 and 1248. Elemental analysis: calcd. value for $C_{62}H_{48}N_2O_4$: 84.14% C, 5.47% H and 3.17% N; found: 84.28% C, 5.39% H and 3.21% N. HRMS (EI) m/z calculated for $C_{62}H_{48}N_2O_4$ $[M]^+$: 884.3614, found: 884.3604.

Synthesis of 2b:

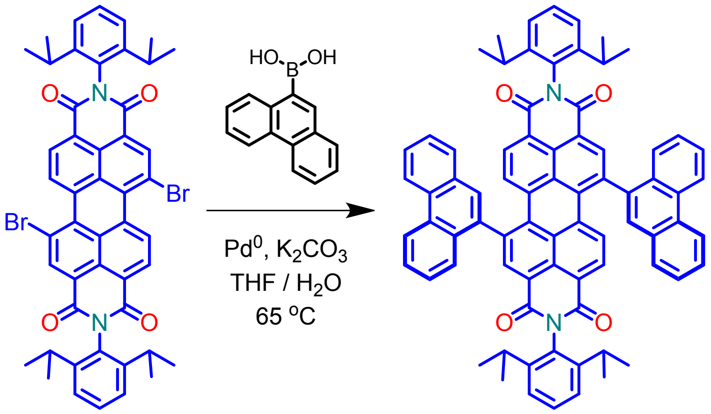

2a **2b**

0.2 mmol of 1,7-dibromoperylenediimide (**2a**) was dissolved in dry THF and 10 mL of 2 M potassium carbonate was added. Reaction mixture was purged with nitrogen for 15 minutes. 0.02 mmol of tetrakis(triphenylphosphine)palladium(0) and 0.50 mmol of 9-phenanthreneboronic acid were added under nitrogen atmosphere. Reaction mixture was refluxed at 65 °C under nitrogen atmosphere. After completion of the reaction, product was extracted in dichloromethane and purified by column chromatography using dichloromethane/petroleum ether mixture (65:35) as eluent. Red coloured solid was obtained as product (**2b**) in 45 % yield. M.p. > 300 °C. ^{1}H NMR [500 MHz, $CDCl_3$, δ]: 8.84 (m, 2H), 8.78 (m, 2H), 8.68 (d, 2H), 8.15 (d, 1H), 8.01 (d, 1H), 7.94 (m, 3H), 7.88 (m, 2H), 7.81 (m, 3H), 7.73 (m, 4H), 7.63 (m, 2H), 7.54 (t, 1H), 7.46 (t, 1H), 7.36 (t, 2H), 7.20 (m, 4H), 2.62 (m, 4H), 1.04 (m, 24H).^{13}C NMR [125 MHz, $CDCl_3$, δ]: 163.69, 163.66, 163.39, 163.33, 145.63, 145.60, 145.50, 139.98, 139.12, 137.53, 135.57, 135.06, 134.96, 134.27, 131.83, 131.71, 131.62, 131.49, 131.44, 130.50, 130.47, 130.39, 129.75, 129.69, 129.07, 128.97, 128.22, 127.76, 127.71, 127.58, 127.55, 127.41, 127.13, 126.56, 125.55, 124.16, 124.07, 123.83, 123.40, 123.34, 123.05, 122.98, 122.48, 122.41, 31.94, 29.71, 29.27, 29.24, 29.13, 29.11, 24.07, 24.00, 23.97, 23.92, 23.91, 22.71 (KBr, cm^{-1}): 2960, 1732, 1694 and 1657.

Elemental analysis: calcd. value for $C_{76}H_{58}N_2O_4$: 85.85% C, 5.50% H and 2.63% N; found: 85.88% C, 5.40% H and 2.65% N. HRMS (EI) m/z calculated for $C_{76}H_{58}N_2O_4$ $[M]^+$: 1062.4397, found: 1062.4367.

Synthesis of 2d

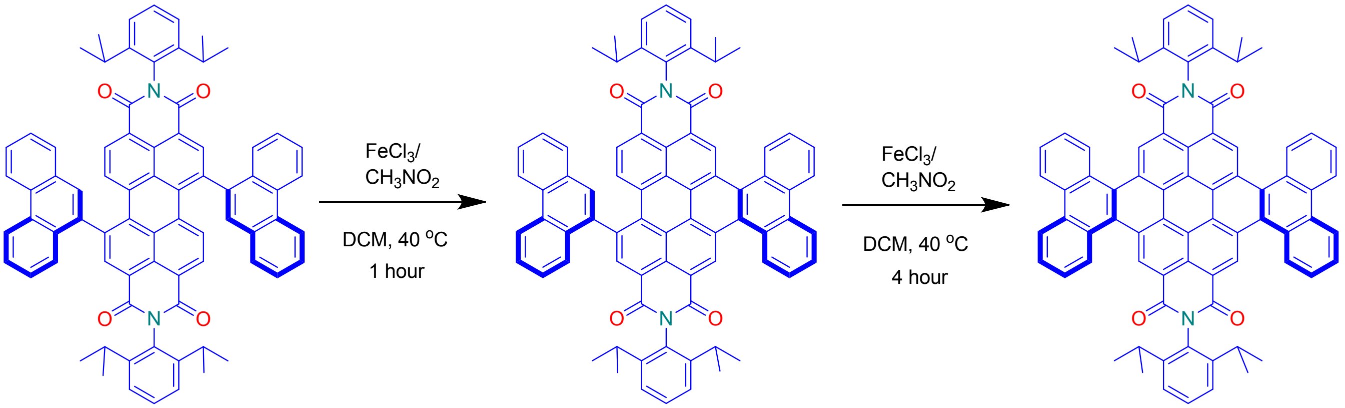

2b **2c** **2d**

100 mg of **2b** was dissolved in 500 mL of dry dichloromethane and heated to 40 °C under nitrogen atmosphere. A solution of 8 equivalents ferric chloride in 10 mL dry nitromethane was added dropwise into the reaction mixture under continuous nitrogen purging. The reaction mixture was refluxed at 40 °C for 1 hour. Aliquot of the reaction mixture was poured into methanol to stop the reaction. Red coloured precipitate was filtered and dried in hot air oven. One side cyclised product **2c** was purified by column chromatographic technique using dichloromethane/petroleum ether (65:35) and characterised by ^1H NMR spectroscopy. M.p. > 300 °C. ^1H NMR [500 MHz, $CDCl_3$, δ]: 10.82 (s, 2H), 8.99 (m, 2H), 8.93 (m, 2H), 7.90 (m, 4H), 7.63 (m, 3H), 7.49 (m, 4H), 7.38 (m, 3H), 7.22 (m, 4H), 7.20 (m, 2H), 7.12 (m, 2H), 3.64 (m, 2H), 2.97 (m, 2H), 1.24 (m, 24H). IR (KBr, cm^{-1}): 1748, 1709 and 1665. HRMS (EI) m/z calculated for $C_{76}H_{56}N_2O_4$ $[M]^+$: 1060.4240, found: 1060.4167. Since, very little material was isolated, further studies were not performed on this molecule. We focused to synthesise both side cyclised poly aromatic imides. Rest of the reaction mixture was refluxed under nitrogen atmosphere for 4 more hours. Then the reaction mixture was poured into methanol and red coloured precipitate was filtered and dried. Product **2d** was purified by silica column chromatographic technique using dichloromethane/petroleum ether (65:35) mixture as eluent. Yield obtained was 40 %. **2d** crystals were obtained from the slow evaporation of toluene solution. M.p. > 300 °C. ^1H NMR [500 MHz, $CDCl_3$, δ]: 10.86 (s, 4H), 8.99 (m, 4H), 8.89 (m, 4H), 7.86 (m, 8H), 7.50 (t, 2H), 7.37 (d, 4H), 2.98 (m, 4H), 1.21 (d, 24H). ^{13}C NMR [125

MHz, $CDCl_3$, δ]: 164.95, 145.87, 131.81, 131.69, 130.77, 129.67, 128.97, 128.43, 128.35, 128.16, 126.88, 124.26, 124.18, 121.25, 29.41, 24.15. IR (KBr, cm^{-1}): 1738, 1701 and 1662. Elemental analysis: calcd. value for $C_{76}H_{54}N_2O_4$: 86.18% C, 5.14% H and 2.64% N; found: 86.08% C, 5.12% H and 2.50% N. HRMS (EI) m/z calculated for $C_{76}H_{54}N_2O_4$ $[M]^+$: 1058.4084, found: 1058.4067.

Synthesis of 3b

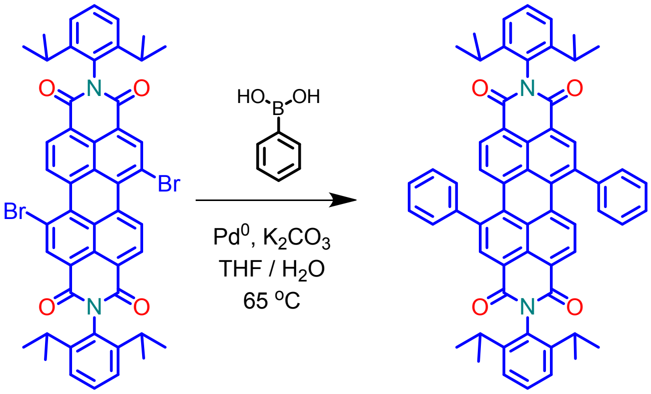

2a **3b**

0.2 mmol of 1,7-dibromoperylenediimide (**2a**) was dissolved in dry THF and 10 mL of 2 M potassium carbonate was added. Reaction mixture was purged with nitrogen for 15 minutes. 0.02 mmol of tetrakis(triphenylphosphine)palladium(0) and 0.50 mmol of phenylboronic acid were added under nitrogen atmosphere. Reaction mixture was refluxed at 65 °C under nitrogen atmosphere. After completion of the reaction, product was extracted in dichloromethane and purified by column chromatography using dichloromethane/petroleum ether mixture (65:35) as eluent. Red coloured solid was obtained as product (**3b**) in 45 % yield. M.p. > 300 °C. ^1H NMR [500 MHz, $CDCl_3$, δ]: 8.66 (s, 2H), 8.15 (d, 2H), 7.87 (d, 2H), 7.57 (m, 4H), 7.46 (m, 8H), 7.27 (d, 4H), 2.84 (m, 4H), 1.12 (d, 24H). ^{13}C NMR [125 MHz, $CDCl_3$, δ]: 163.10, 162.50, 144.64, 141.12, 140.44, 134.83, 134.37, 132.01, 129.46, 129.30, 128.99, 128.63, 128.07, 127.79, 127.43, 123.09, 121.34, 120.96, 28.17, 23.04, 22.97. IR (KBr, cm^{-1}): 2960, 1732, 1694 and 1657. Elemental analysis: calcd. value for $C_{60}H_{50}N_2O_4$: 83.50% C, 5.84% H and 3.25% N; found: 83.62% C, 5.60% H and 3.35% N. HRMS (EI) m/z calculated for $C_{60}H_{50}N_2O_4$ $[M]^+$: 862.3771, found: 862.3777.

Synthesis of 3c

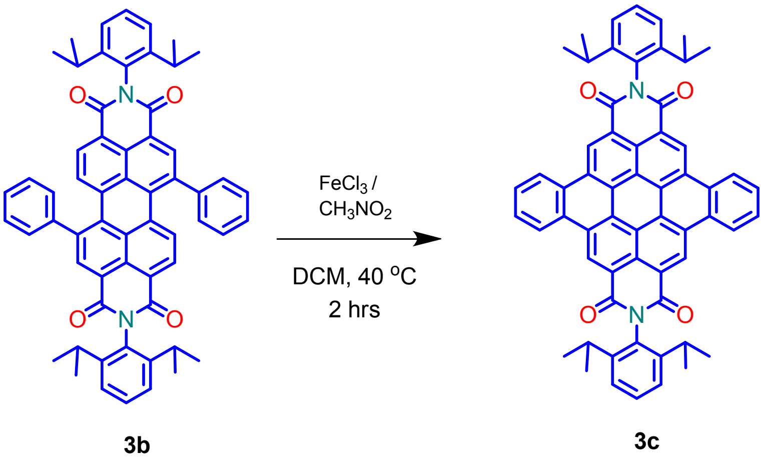

100 mg of **3b** was dissolved in 500 mL of dry dichloromethane and heated to 40 °C under nitrogen atmosphere. A solution of 8 equivalents ferric chloride in 10 mL dry nitromethane was added dropwise into the reaction mixture under continuous nitrogen purging. The reaction mixture was refluxed at 40 °C for 1 hour. Reaction temperature was limited by the boiling point of dichloromethane. Completion of the reaction is monitored through thin layer chromatographic (TLC) technique. Then methanol was poured into the reaction mixture to stop the reaction. Orange coloured product was precipitated. **3c** was purified by silica column chromatographic technique using dichloromethane/petroleum ether (60:40) mixture as eluent. Yield obtained was 50 %. M.p. > 300 °C. ^{1}H NMR [500 MHz, $CDCl_3$, δ]: 10.68 (s, 4H), 9.56 (m, 4H), 8.17 (m, 4H), 7.53 (t, 2H), 7.40 (d, 4H), 2.94 (m, 4H), 1.15 (d, 24H). ^{13}C NMR [125 MHz, $CDCl_3$, δ]: 164.20, 146.47, 131.70, 130.12, 129.83, 129.75, 129.37, 125.30, 125.20, 124.90, 124.82, 124.40, 124.06, 122.65, 29.75, 24.19. IR (KBr, cm^{-1}): 2950, 1715, 1680, 1596 and 1458. Elemental analysis: calcd. value for $C_{60}H_{46}N_2O_4$: 83.89% C, 5.40% H and 3.25% N; found: 83.98% C, 5.29% H and 3.21% N. HRMS (EI) m/z calculated for $C_{62}H_{48}N_2O_4$ $[M]^+$: 858.3458, found: 858.3444.

Table S1. A comparative account of the ISC properties (ΔE_{ST}, Φ_T and τ_{ISC}) of allotropes of carbon in various literature. Our data correspond to the contorted aromatics 1c and 2d.

Molecule	Structure	ISC Parameter	Reference
Fullerene[7] 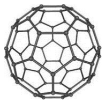	C_{60}	ΔE_{ST} =9 kcal/mol; $\Phi_T \sim 1$; $\tau_{ISC} = 7 \text{ X } 10^8 \text{ s}^{-1}$	*CRC Handbook of Organic Photochemistry and Photobiology, Volumes 1 & 2, 2nd Edition*, **2003**.
	C_{70}	$\Delta E_{ST} \sim 9$ kcal/mol; Φ_T =0.9; $\tau_{ISC} = 8 \text{ x } 10^8 \text{ s}^{-1}$	
	C_{76}	ΔE_{ST} =n.d. Φ_T =0.05;τ_{ISC}= 6.0 x 10^8 s^{-1}	*J. Phys. Chem. A* **1997,** 101, 4840-4844
	C_{82}	ΔE_{ST} =n.d.; $\Phi_T \sim 0.01$; τ_{ISC} = n.d.	*J. Phys. Chem. B* **1999**, 103, 9519-9523
Carbon Nanotube	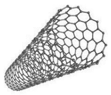	ΔE_{ST} =8 kcal/mol; Φ_T = n.d.; τ_{ISC} = 5.0 x 10^{10} s^{-1}	*J. Am. Chem. Soc.* **2011**, 133, 17156–17159
Carbon Nanohoop	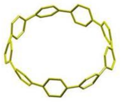	ΔE_{ST} =9 kcal/mol; Φ_T = 0.18; τ_{ISC} = n.d.	J. Phys. Chem. A, **2014**, *118*,1595–1600
Sumanene	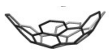	ΔE_{ST} = n.d. Φ_T = n.d.; τ_{ISC} = n.d.	*Chem. Eur. J.* **2014**, *20*, 598 – 608
Carbon Nanohorn	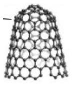	ΔE_{ST} = n.d. Φ_T = n.d.;τ_{ISC} = 1.3 x 10^{11} s^{-1}	*Chem. Sci.,* **2014,** *5*, 2072-2080
Graphene	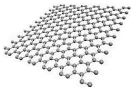	$\Delta E_{ST} \sim 0$ $\Phi_T \sim 0$; τ_{ISC} = n.d.	*Phys. Rev. Lett.* **2009**, *103,* 026804
Graphene Quantum Dot	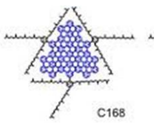	ΔE_{ST} =4 kcal/mol; Φ_T = n.d.; τ_{ISC} = n.d.	*Nano Lett.* **2010,** *10*, 2679–2682
Grossly Warped Nanographene	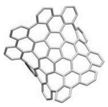	ΔE_{ST} = n.d. Φ_T = n.d.; τ_{ISC} = n.d.	*Nat. Chem.,* **2013**, *5,* 739-744
Graphene Nanoribbon	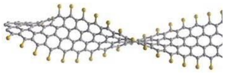	ΔE_{ST} =n.d. Φ_T = n.d.; τ_{ISC} = n.d.	*J. Am. Chem. Soc.* **2014**, 136, 8122-8130
Di-PDI	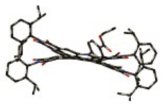	ΔE_{ST} =n.d. Φ_T = 0.91.; τ_{ISC} = 2 x 10^9 s^{-1}	*J. Phys. Chem. Lett.* **2010,** 1, 2499-2502.

Contorted Aromatics	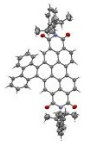	ΔE_{ST} ~ 8.5 kcal/mol<br>Φ_T = 0.10; τ_{ISC} = 1 x 10^9 s^{-1}	Our data
	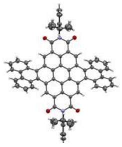	ΔE_{ST} ~ 8.4 kcal/mol<br>Φ_T = 0.30; τ_{ISC} = 4 x 10^{10} s^{-1}	Our data

*n.d. – not determined

Table S2. Single crystal analysis of 1c and 2d

	**1c**	**2d**
formula	$C_{62}H_{48}N_2O_4$	$C_{76}H_{54}N_2O_4$
formula wt	885.0555	1059.2530
colour, shape	Red, block	Red, Block
crystal system	Tetragonal	Triclinic
space group,	P-4_3	P-1
a, Å	13.9364(2)	11.4627(5)
b, Å	13.9364(2)	11.8704(5)
c, Å	34.5285(7)	15.6450(6)
α, deg	90	76.067(2)
β, deg	90	89.834(2)
γ, deg	90	72.770(10)
V, $Å^3$	6706.2(2)	1968.03(14)
temp, K	296	296
d_{calcd}, g/cm^{-3}	1.348	1.205
no. of reflections collected	11487	7652
no. of unique reflections	9920	3794
$2\theta_{max}$, deg	50	50
no. of parameters	803	472
R1, wR2 (I > 2σ(I))	0.1013, 0.0730	0.1469, 0.0694
R1, wR2 (all data)	0.2326, 0.2046	0.2099, 0.1717
goodness of fit	1.022	1.025
CCDC number	1402604	1402605

Table S3. Electrochemical properties of the derivatives 1c, 2d and 3c

Derivative	E_{red1} (V)	E_{red2}(V)

1c	-0.69	-0.92
2d	-0.79	-1.01
3c	-0.55	-0.79

Table S4. Photophysical properties of the derivatives 1c, 2d and 3c

	Solution (Toluene)						
	λ_{abs}, nm	λ_{fl}, nm	Φ_F	τ_{fl}, ns	φ_T	τ_T, μs	τ_{ISC}, ps
1c	475, 505, 545	555, 597, 652	0.70	10	0.1	3.7	22
2d	385, 416, 445 475, 539, 582	586, 635, 696	0.40	5.4	0.3	19.6	19
3c	460, 490	510, 550, 580	0.85	5.5	0	--	--

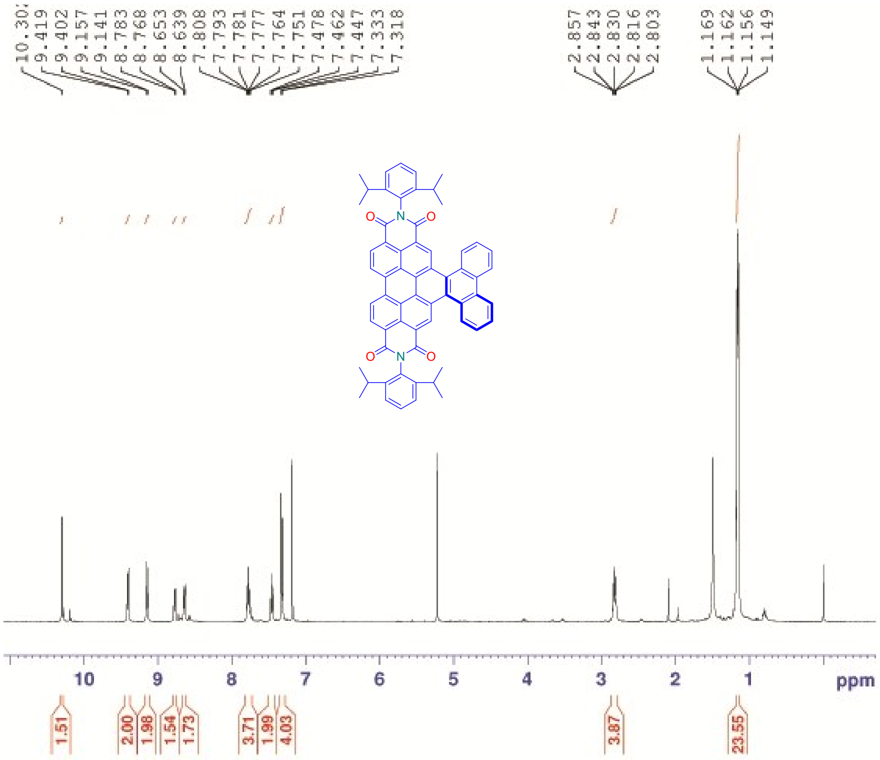

Figure S1. ^{1}H NMR spectrum of compound 1c.

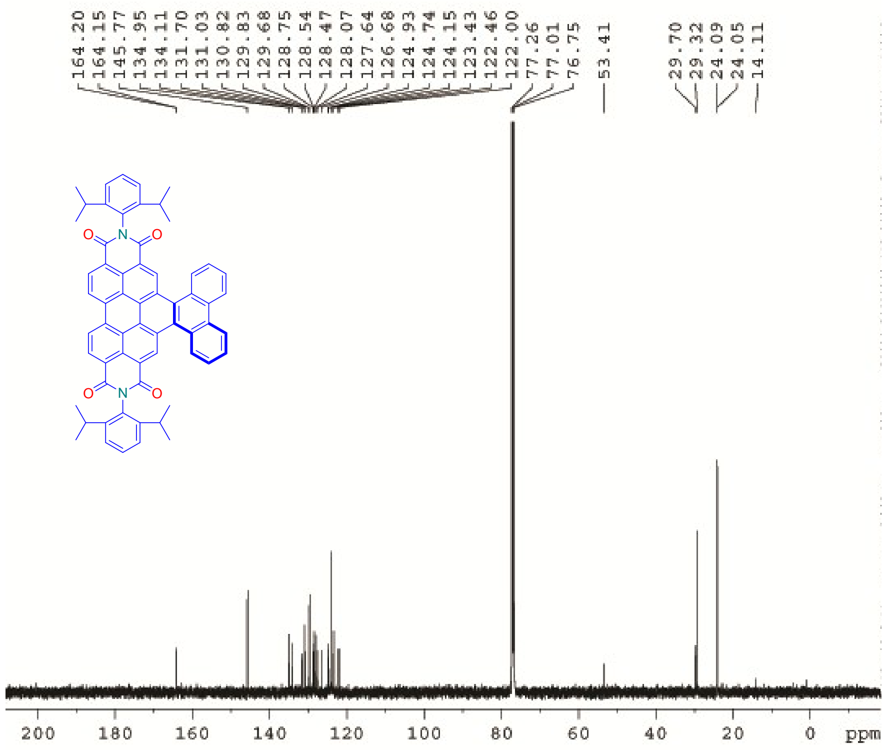

Figure S2. ^{13}C NMR spectrum of compound 1c.

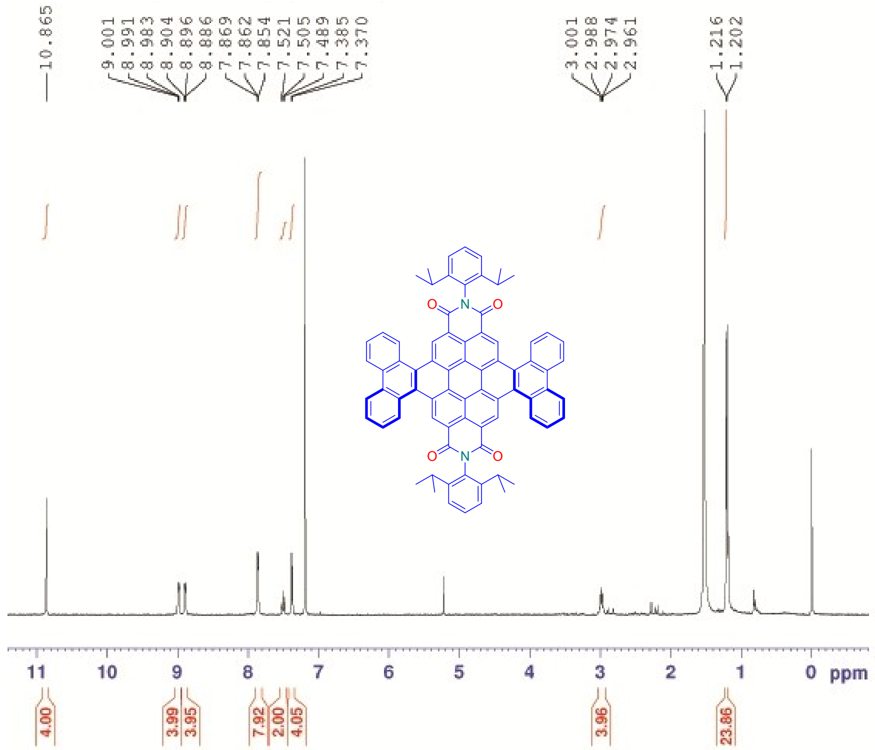

Figure S3. ^{1}H NMR spectrum of compound 2d.

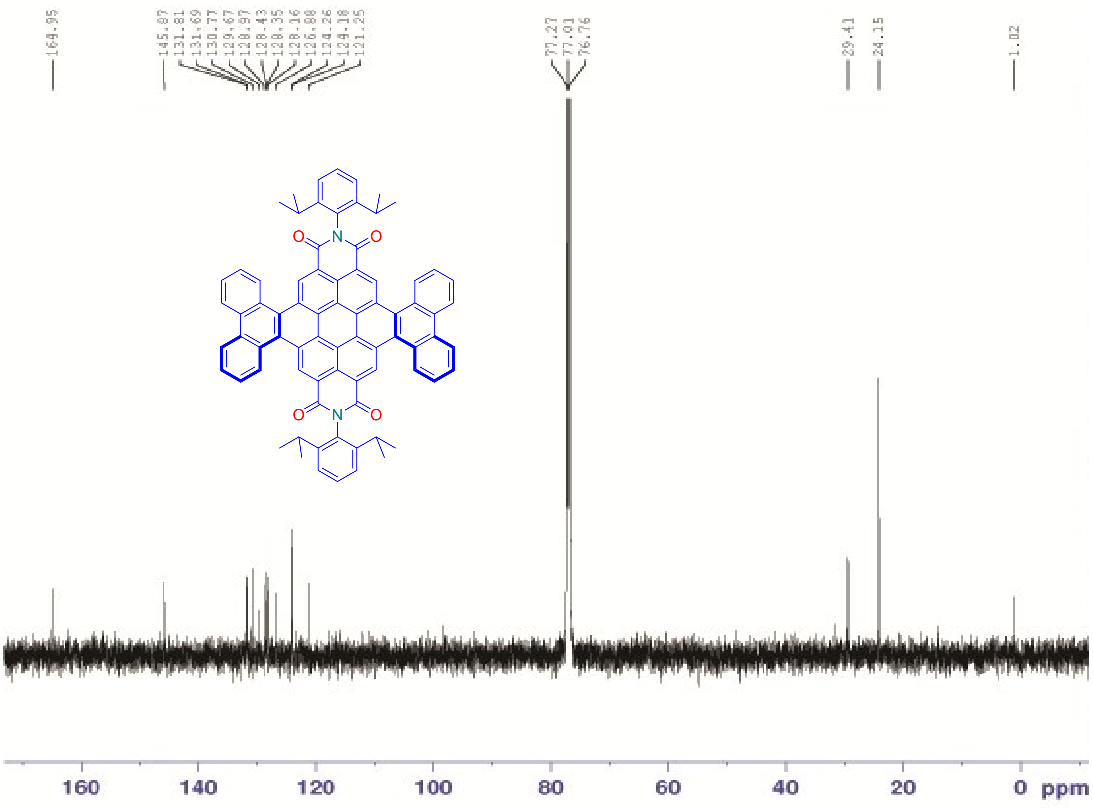

Figure S4. ^{13}C NMR spectrum of compound 2d.

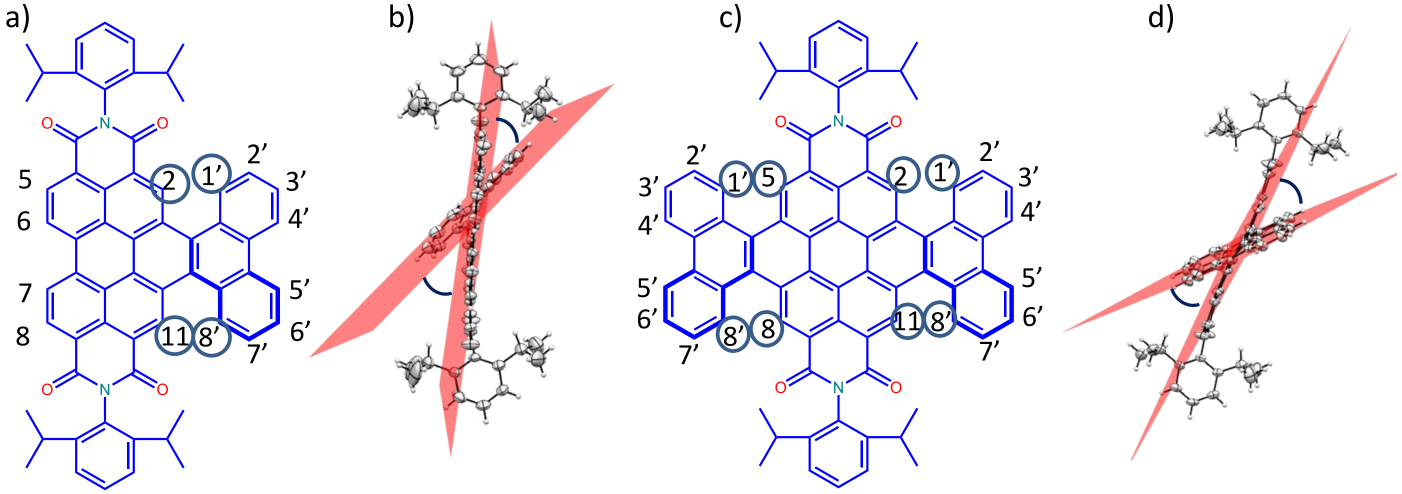

Figure S5. Chemical structures displaying H•••H repulsion in a) 1c and c) 2d; Schematic representation showing the perylenediimide and phenanthrene planes in the crystal structure of b) 1c and d) 2d.

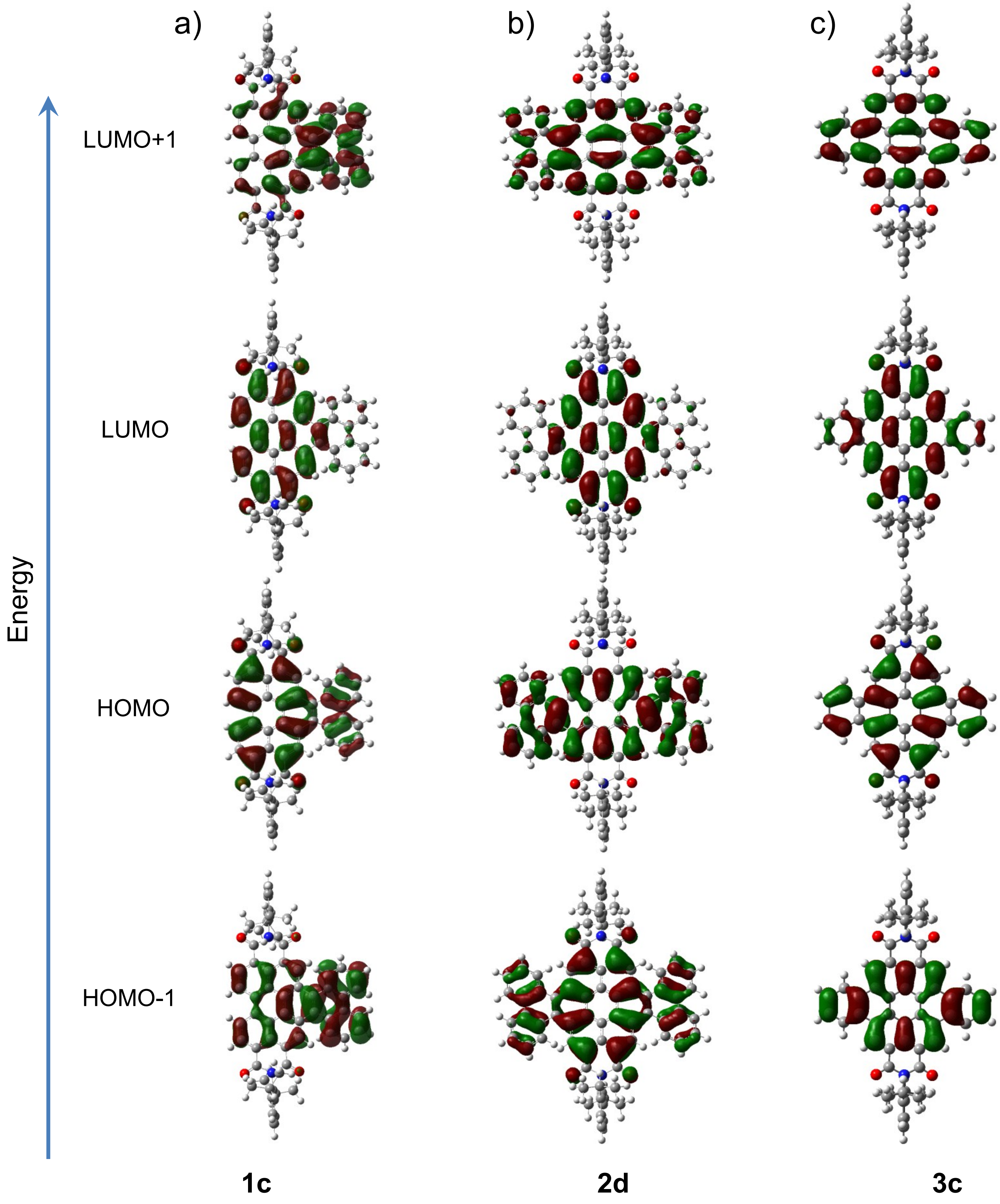

Figure S6. Frontier molecular orbital diagram of a) 1c, b) 2d and c) the model derivative 3c

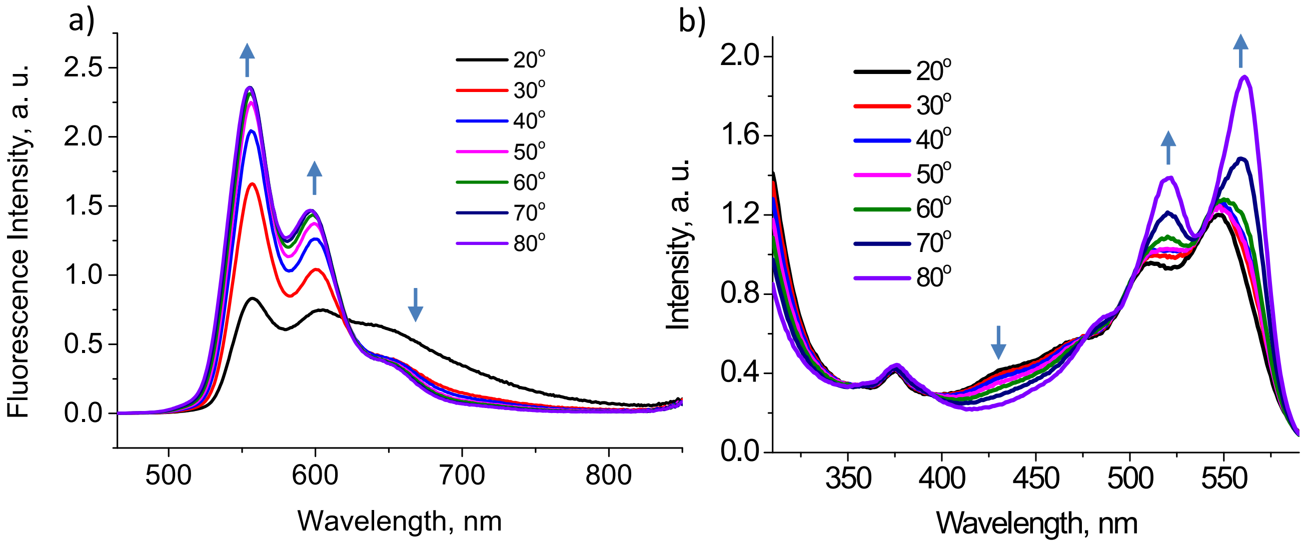

Figure S7. a) Temperature dependent emission spectrum (λ_{ex}=480 nm) b) temperature dependent excitation spectrum (λ_{em}=600 nm) of dilute solution (1 x 10^{-6} M) of 1b in toluene.

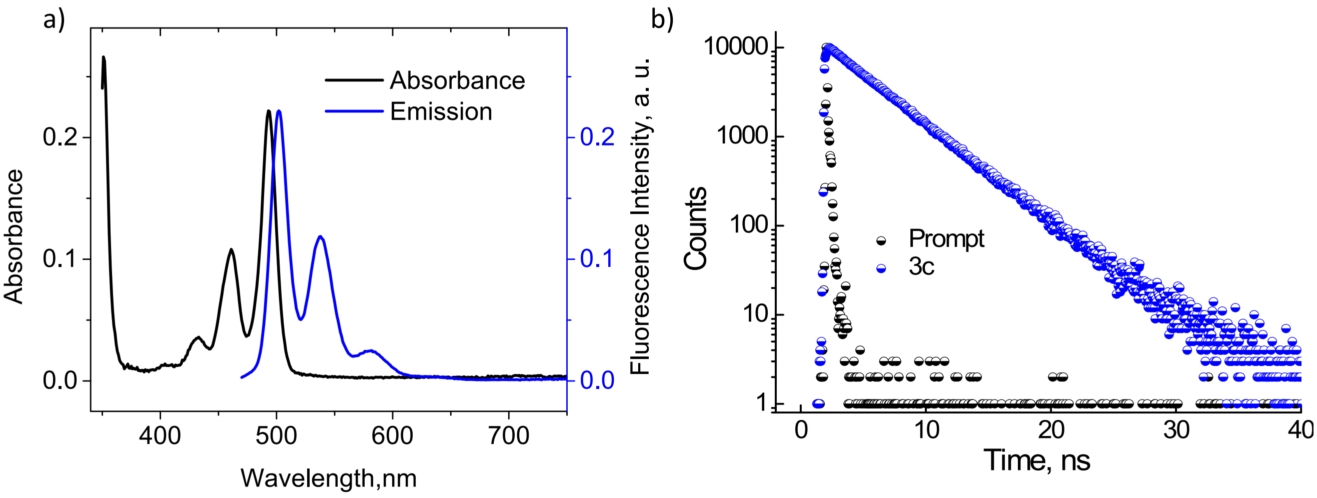

Figure S8. a) Absorption and emission (λ_{ex}=480 nm) spectra; b) time dependent fluorescence spectrum (λ_{ex}=480 nm and collected at 550 nm) of the model derivative 3c in toluene.

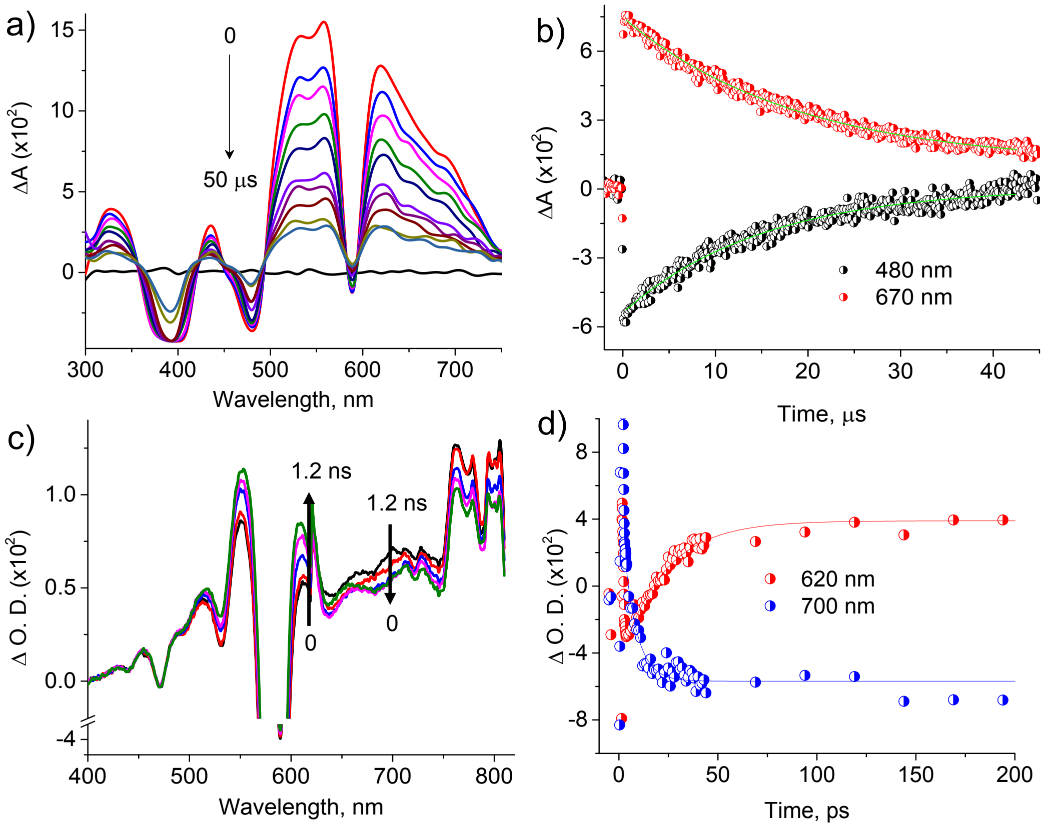

Figure S9. a) nTA spectra (λ_{ex}=355 nm); b) corresponding kinetics plots of excited singlet and triplet states in toluene; c) fTA spectra (λ_{ex}=300 nm) and d) corresponding decay and bleach recovery profile of the compound 2d in toluene.

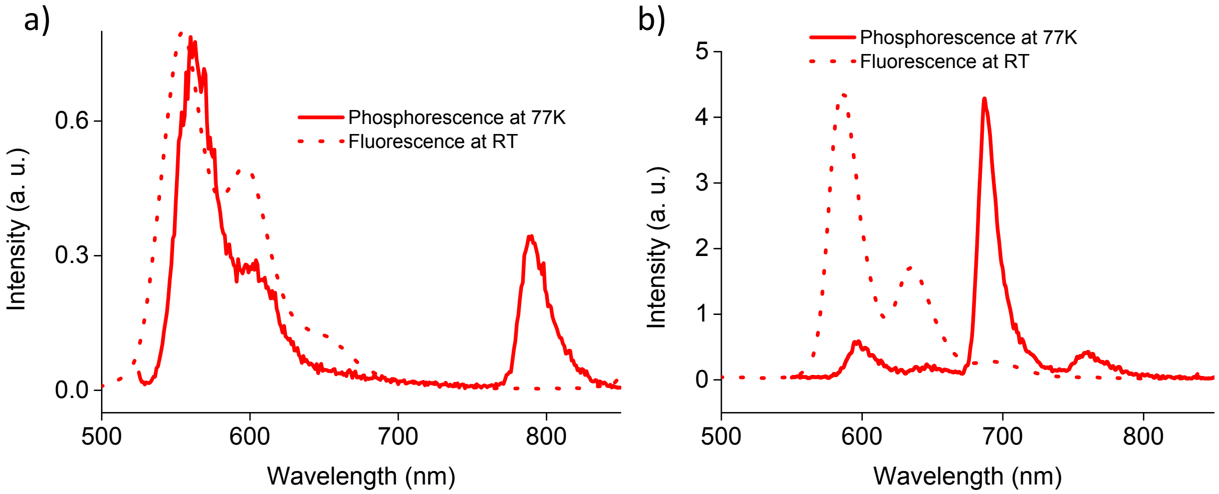

Figure S10. Normalized fluorescence spectra in DCM at RT and phosphorescence spectra in DCM-EtOH-CH_3I (1: 1: 0.1) glass at 77K of a) 1c and b) 2d.

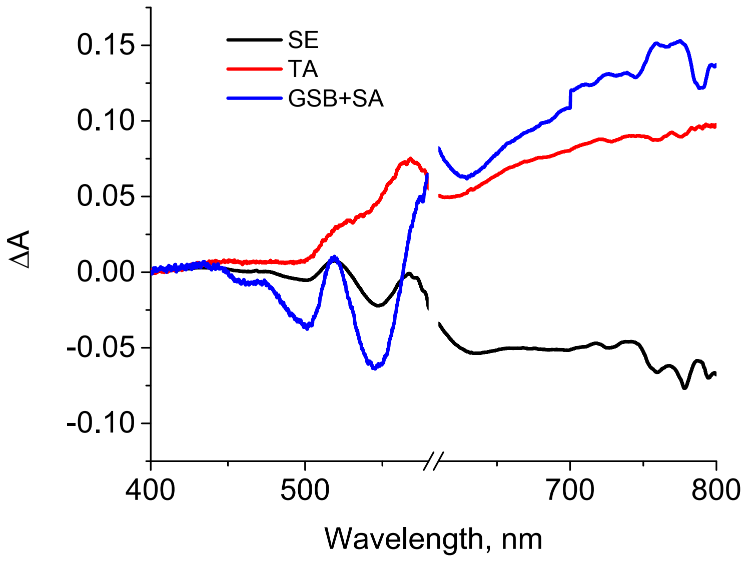

Figure S11. fTA spectra of 1c in toluene (λ_{ex} = 300 nm) obtained from the SVD analysis (SE- stimulated emission; TA-triplet absorption; GSB - ground state bleach.

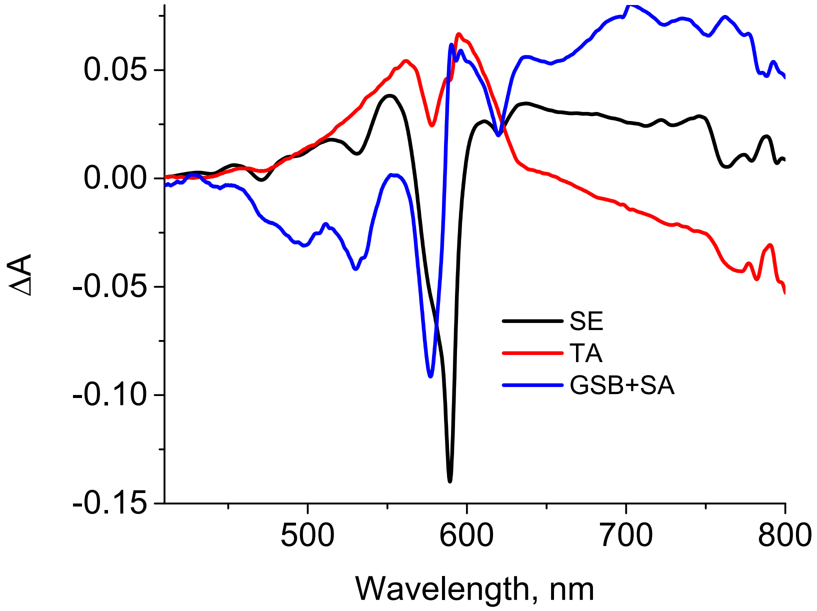

Figure S12. fTA spectra of 2d in toluene (λ_{ex} = 300 nm) obtained from the SVD analysis (SE- stimulated emission; TA-triplet absorption; GSB - ground state bleach.

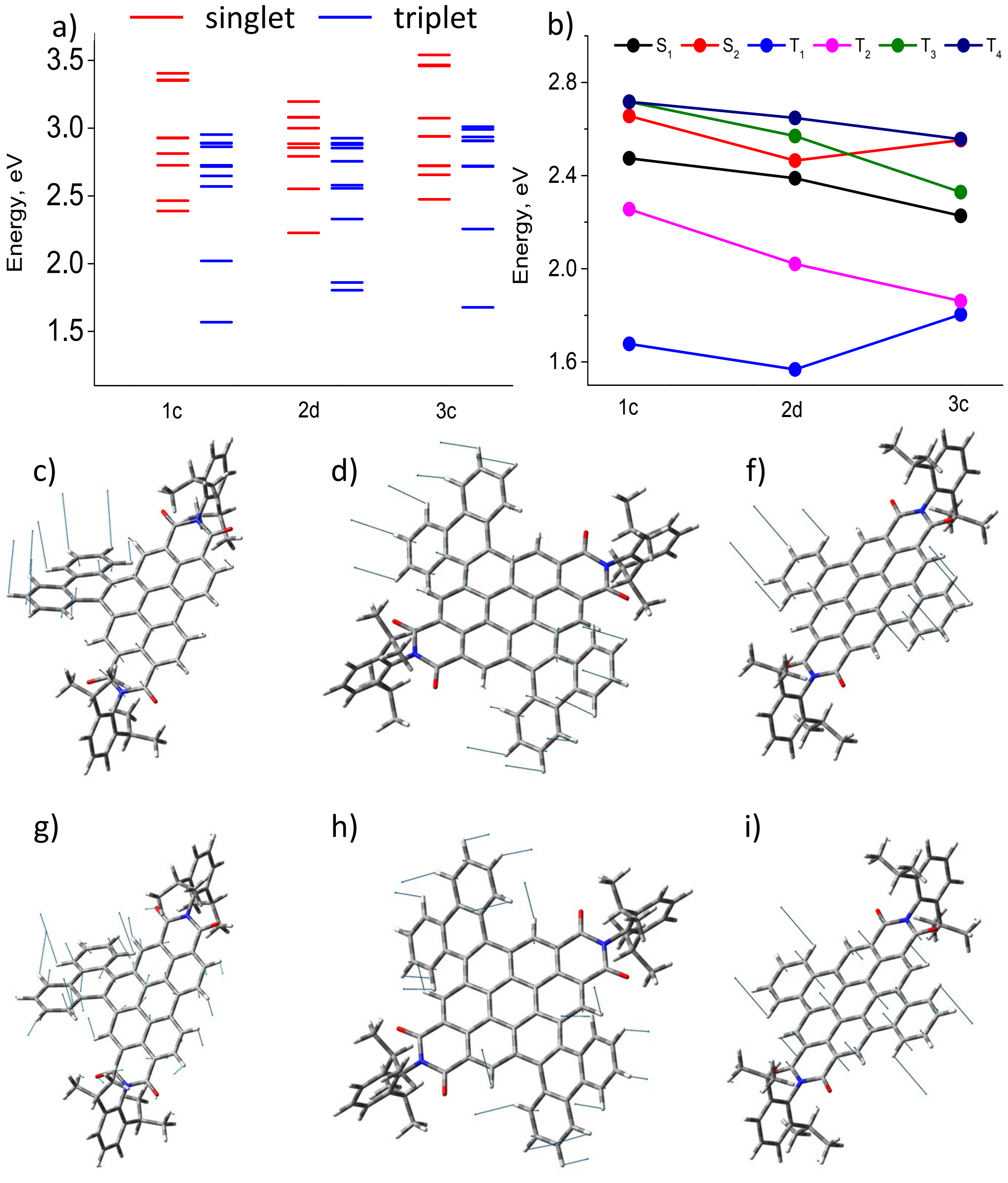

Figure S13. a) Singlet and triplet energy levels of 1c, 2d and 3c calculated from TD-DFT at the basis set of BYLYP/6-311G++(d,p); b) comparison of low-lying singlet and triplet energy levels in the

derivatives; c), d) and f) out of plane mode C-H bending in 1c, 2d and 3c respectively; g), h) and i) out of plane mode C=C bending in 1c, 2d and 3c respectively.

References:

1. D. F. Eaton, *Pure Appl. Chem.*, 1988, **60**, 1107-1114.
2. W. E. Ford and P. V. Kamat, *J. Phys. Chem.*, 1987, **91**, 6373-6380.
3. J. J. Snellenburg, S. P. Laptenok, R. Seger, K. M. Mullen and I. H. van Stokkum, *J. Stat. Softw.*, 2012, **49**, 1--22.
4. Y. Wu, Y. Zhen, Y. Ma, R. Zheng, Z. Wang and H. Fu, *J. Phys. Chem. Lett.*, 2010, **1**, 2499-2502.
5. T. Hinoue, Y. Shigenoi, M. Sugino, Y. Mizobe, I. Hisaki, M. Miyata and N. Tohnai, *Chem. Eur. J.*, 2012, **18**, 4634-4643.
6. Gaussian 09, M. J. Frisch, G. W. Trucks, H. B. Schlegel, G. E. Scuseria, M. A. Robb, J. R. Cheeseman, G. Scalmani, V. Barone, B. Mennucci, G. A. Petersson, H. Nakatsuji, M. Caricato, X. Li, H. P. Hratchian, A. F. Izmaylov, J. Bloino, G. Zheng, J. L. Sonnenberg, M. Hada, M. Ehara, K. Toyota, R. Fukuda, J. Hasegawa, M. Ishida, T. Nakajima, Y. Honda, O. Kitao, H. Nakai, T. Vreven, J. A. Montgomery Jr., J. E. Peralta, F. Ogliaro, M. J. Bearpark, J. Heyd, E. N. Brothers, K. N. Kudin, V. N. Staroverov, R. Kobayashi, J. Normand, K. Raghavachari, A. P. Rendell, J. C. Burant, S. S. Iyengar, J. Tomasi, M. Cossi, N. Rega, N. J. Millam, M. Klene, J. E. Knox, J. B. Cross, V. Bakken, C. Adamo, J. Jaramillo, R. Gomperts, R. E. Stratmann, O. Yazyev, A. J. Austin, R. Cammi, C. Pomelli, J. W. Ochterski, R. L. Martin, K. Morokuma, V. G. Zakrzewski, G. A. Voth, P. Salvador, J. J. Dannenberg, S. Dapprich, A. D. Daniels, Ö. Farkas, J. B. Foresman, J. V. Ortiz, J. Cioslowski and D. J. Fox, Wallingford, CT, USA, 2009.
7. W. M. Horspool and F. Lenci, *CRC Handbook of Organic Photochemistry and Photobiology, Volumes 1 & 2, Second Edition*, CRC Press, 2003.
